# Orbital angular momentum-mediated machine learning for high-accuracy mode-feature encoding

**DOI:** 10.1038/s41377-024-01386-5

**Published:** 2024-02-14

**Authors:** Xinyuan Fang, Xiaonan Hu, Baoli Li, Hang Su, Ke Cheng, Haitao Luan, Min Gu

**Affiliations:** 1 Institute of Photonic Chips, University of Shanghai for Science and Technology, Shanghai, 200093 China; 2 Centre for Artificial-Intelligence Nanophotonics, School of Optical-Electrical and Computer Engineering, University of Shanghai for Science and Technology, Shanghai, 200093 China

**Keywords:** Applied optics, Optical techniques

## Abstract

Machine learning with optical neural networks has featured unique advantages of the information processing including high speed, ultrawide bandwidths and low energy consumption because the optical dimensions (time, space, wavelength, and polarization) could be utilized to increase the degree of freedom. However, due to the lack of the capability to extract the information features in the orbital angular momentum (OAM) domain, the theoretically unlimited OAM states have never been exploited to represent the signal of the input/output nodes in the neural network model. Here, we demonstrate OAM-mediated machine learning with an all-optical convolutional neural network (CNN) based on Laguerre-Gaussian (LG) beam modes with diverse diffraction losses. The proposed CNN architecture is composed of a trainable OAM mode-dispersion impulse as a convolutional kernel for feature extraction, and deep-learning diffractive layers as a classifier. The resultant OAM mode-dispersion selectivity can be applied in information mode-feature encoding, leading to an accuracy as high as 97.2% for MNIST database through detecting the energy weighting coefficients of the encoded OAM modes, as well as a resistance to eavesdropping in point-to-point free-space transmission. Moreover, through extending the target encoded modes into multiplexed OAM states, we realize all-optical dimension reduction for anomaly detection with an accuracy of 85%. Our work provides a deep insight to the mechanism of machine learning with spatial modes basis, which can be further utilized to improve the performances of various machine-vision tasks by constructing the unsupervised learning-based auto-encoder.

## Abstract (web summary)

The theoretical unlimited orbital angular momentum states have been utilized as node signals in an optical neural network to implement machine learning tasks.

## Graphical abstract

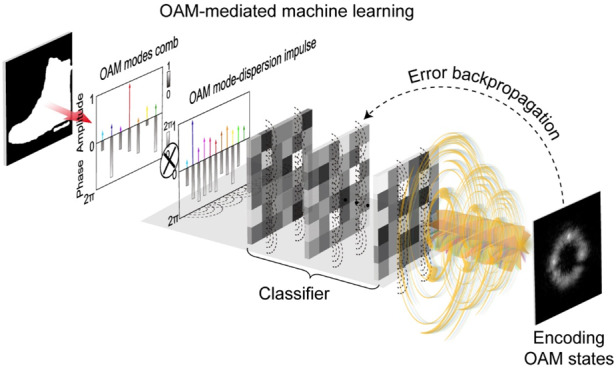

## Introduction

Artificial neural networks (ANNs) provide a mathematical model that emulates the brain function for machine learning^1^, which can be performed in various physical domains^2^, such as electronics, optics and mechanics. To dramatically improve computing speed and energy efficiency^3^, various photonic computing approaches have been proposed to construct optical neural networks (ONNs), wherein different properties of light (e.g. time^4^, space^5^, wavelength^6^, polarization^7^) could be utilized for photonic multiplexing to achieve high parallelism, large-data throughput and large-scale interconnectivity. As another unique degree of freedom of light, the orbital angular momentum (OAM) division^8–10^ with unlimited orthogonal states could be utilized to convey information, creating the concepts of digital spiral imaging^11^, high-capacity optical communications^12^, optically addressable video holography^13–15^ and display^16^, six-dimensional data storage^17^, spatiotemporal light fields^18^, and high-dimensional quantum entanglement^19^. However, OAM has never been adopted to represent the signal of the input/output nodes in the neural network model.

**Fig. 1:**
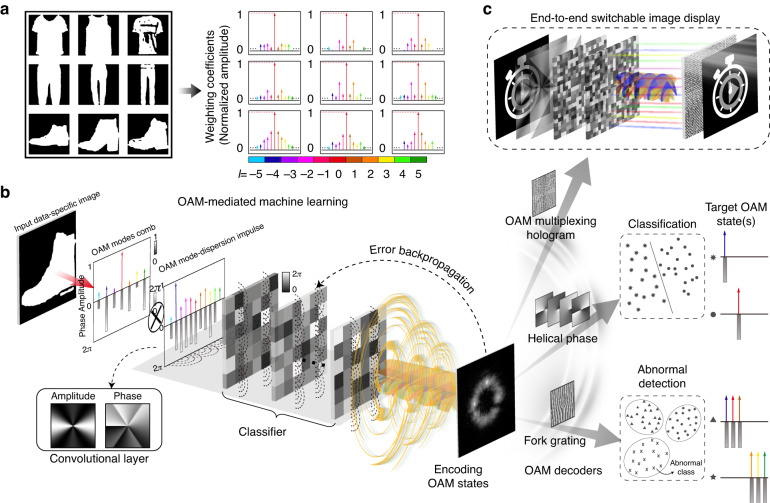
Conceptual illustration of the OAM-mediated machine learning and the application of all-optical information mode-feature encoding. **a** OAM mode combs with normalized weight coefficients of the data-specific images. The pseudo-colors represent different OAM orders (*l*). **b** The architecture of the all-optical CNN for OAM-mediated machine learning, which can be applied to encode a data-specific image into OAM states. The photonic neural network comprises a trainable convolutional layer which can provide an OAM mode-dispersion impulse to densify the input OAM mode comb and extract the feature, and successive phase-engineered diffractive layers with finite size as a classifier to reduce the dense OAM mode spectrum to a couple of target terms due to the OAM mode-dispersion selectivity. **c** The proposed CNN with an appropriate OAM modes decoder can be applied in image classification, end-to-end switchable image display, and all-optical abnormal detection, respectively. Due to the weighting coefficients of the target OAM states are set as amplitude only (without phase differences), only the energy weighting coefficients of the output OAM spectrum terms are needed to be detected in the last two machine leaning tasks

Photonic matrix-vector operations on various physical dimensions are necessary to provide the fundamental building block for ONNs. As such, to physically interpret OAM information as matrix-vector of ONNs, the input raw data in the space domain should be transformed into indistinguishable and large OAM mode combs with most non-zero amplitude coefficients terms concentrating on the low-order OAM mode components (Fig. 1a)^11,20^, which indicates sparse OAM information feature with underlying commonalities. Notably, the straightforward optical matrix operations in the OAM domain are inaccessible^21^, thus imposing a fundamental challenge of extracting the OAM mode-feature and reducing redundant or irrelevant mode components determined by further end’s applications. As such, although various micron- and nanophotonic devices designed by physical rules have been introduced to generate and detect OAM states with helical wavefronts^22–30^, neither waveguide-based ONNs^5,6,31,32^ nor diffraction-based ONNs^7,33–35^ are capable for OAM-mediated machine learning because physically these systems are lack of the OAM selectivity at a micro-scale.

Inspired by the biological behavior of the visual cortex system, convolutional neural networks (CNNs) have been put forward to enhance the prediction accuracy through inserting a convolution-based feature extraction block before classifying^36^. Specifically, the parametric complexity of high-dimensional data can be greatly reduced by CNN after abstracting the features of input data in their raw form. To construct an all-optical CNN for OAM-mediated machine learning, it is necessary to further present an OAM mode selectivity when dispersing the OAM mode combs. As a specific OAM mode featuring amplitude distribution described by the Laguerre-Gaussian (LG) polynomials^37^, LG modes are capable to fully represent the spatial structure of a transverse field, which has been applied in face recognition^38^ and rotational object imaging^39^. Physically, LG modes are the eigen solutions of the paraxial Helmholtz equation, which inherently results in the distinctive diffraction losses when they diffract by a finite-sized object^40^. Thus unique physical feature gives us the ground to exploit the OAM mode-dispersion selectivity through diffractive elements with finite size^41^.

Here, we demonstrate an entirely new concept of OAM-mediated machine learning with an all-optical CNN, and further apply it in all-optical information mode-feature encoding. The architecture of the CNN for OAM-mediated machine learning is illustrated in Fig. 1b. After expansion of the input object into OAM mode combs with complex-amplitude weighting coefficients, a trainable OAM mode-dispersion impulse has been adopted to provide a convolution operation in the OAM domain to densify the input OAM mode combs and extract the mode-features. Here, the OAM mode-dispersion impulse is the OAM expansion of a complex-amplitude layer. To further achieve mode-feature encoding for specific applications, it is necessary to reduce the dense OAM mode combs to a couple of target terms, which can be seen as the compression of OAM mode-features. As such, due to different diffraction losses of LG modes, the successive phase-engineered diffractive layers with finite size are utilized to implement mode conversion in the LG mode basis, which can achieve OAM mode-dispersion selectivity. Notably, through controlling the amplitude coefficients of the output OAM mode states, the CNN designed by error backpropagation algorithm can distribute the dominant energy on targeted OAM states constituting the output electrical field for encoding. Finally, the CNN as an encoder, associated with an appropriate OAM-dependent hologram as a decoder, can be applied in various applications as shown in Fig. 1c. Specifically, when the OAM-multiplexing hologram is applied in decoding, different holographic images can be reconstructed by different encoded OAM beams^15^, resulting in an end-to-end switchable image display. Moreover, helical phase plates or fork gratings can be designed to obtain the energy weighting coefficients of the output OAM mode comb components as OAM spectrum measurement. For the application of classification, we only need to find each OAM terms with the dominant energy weighting coefficients to identify the input symbols. More significantly, the energy weighting coefficients of the multiplexed OAM states could be utilized to represent the original images, providing an all-optical dimension reduction method for improving the efficiency and robustness of abnormal detection.

## Results

### Design principle of the CNN

**Fig. 2:**
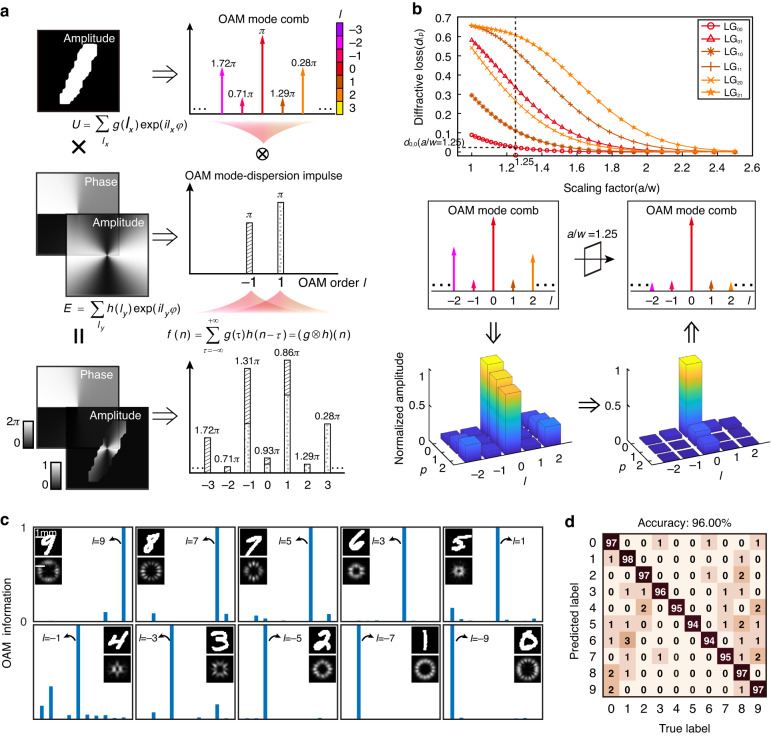
Physical principles of the CNN for mode-feature encoding. **a** Illustration of a convolution operation of an OAM mode comb with an OAM mode-dispersion impulse based on superposed electrical fields in the spatial domain. **b** Diffraction losses of LG modes and the evolution of the OAM mode combs due to a single diffractive layer with finite size. **c** Encoding MNIST database into ten OAM modes based on the CNN. For ten blinding testing images, the intensity distributions and the OAM information are shown, respectively. **d** The confusion matrix with a testing encoding accuracy of 96.0%

The physical principles of the all-optical CNN, including an OAM mode-dispersion impulse and an OAM mode-dispersion selectivity, are illustrated in Fig. 2. To realize a common optical convolution in the spatial domain, a classical 4-*f* optical setup is needed to superpose the electrical fields in the spatial frequency domain wherein the lens is utilized to perform Fourier transformation^42^. In comparison, the superposed electrical fields in the spatial domain results in the convolution of an OAM mode comb with an OAM mode-dispersion impulse (Fig. 2a). For example, the electrical field *U* of a handwrite digit “1” can be decomposed into the complex-amplitude OAM mode comb *g*(*l*_*x*_) through $U={\sum }_{{l}_{x}}{\text{g}}({l}_{x})\,\exp ({\text{i}}{l}_{x}\varphi )$. When imprinted on the complex-amplitude field *E* comprising two OAM mode components *l*_*y*_ = −1 and 1, the complex-amplitude weight coefficient of each OAM mode component *l*_*x*_ is multiplied by the OAM mode-dispersion impulse *h*(*l*_*y*_) provided by the complex-amplitude field $E={\sum }_{{l}_{y}}h({l}_{y})\exp (i{l}_{y}\varphi )$ and then superposed. As such, it can be mathematically defined as a one-dimensional convolution operation and the OAM mode-dispersion impulse *h*(*l*_*y*_) is denoted as the convolution kernel (Supplementary Note 1)^43^. Notably, the convolution with an OAM mode-dispersion impulse provides an effective method to increase the limited OAM mode comb components with non-zero amplitude coefficients (Supplementary Fig. S1), laying the physical foundation to extract the features of input OAM mode combs in machine learning.

Next, the diffraction losses of distinctive LG modes are analyzed to illustrate the capability to control the evolution of the OAM mode combs (Fig. 2b). Here, the diffraction loss δ_*l,p*_ is defined as the reduced amplitude of LG_*l,p*_ mode (the OAM order is *l* and the radial index is *p*) caused by the finite size of the diffractive layer. Without losing generality, the model of a single diffractive layer is adopted (Supplementary Note 2). As a result, the relationship between scaling factor a/w, and the diffraction losses is given for LG_0,0_, LG_0,1_, LG_1,0_, LG_1,1_, LG_2,0_, and LG_2,1_. Here, 2a and w represents the width of the diffractive layer and the beam waist, respectively. As such, the evolution of the OAM mode combs can be further obtained after decomposing them into the LG mode basis (Supplementary Note 3, Supplementary Fig. S2). For example, as shown in the lower panel of Fig. 2b, the diffraction loss of the LG mode basis of the handwrite digit “1” is given, and the normalized amplitude of the OAM mode component with *l* = 0 improves remarkably (Supplementary Note 4). It can be concluded that an OAM mode-dispersion selectivity can be achieved for the diffractive systems with finite size. Then the OAM mode comb can be converted into the target OAM state through the mode conversion by the deep-learning based diffractive layers, in which the LG mode basis is adopted as the target label. Here, we verified the analysis above by experimentally converting a specific category of images with various squatting poses into LG_4,0_ through two cascaded diffractive layers with finite size, wherein a helical phase featuring dominant OAM mode component with *l* = 4 for mode conversion appears in the second diffractive layer (Supplementary Note 5, Supplementary Fig. S3).

To achieve high-accuracy information mode-feature encoding, harnessing the OAM mode-dispersion impulse for feature extraction and the OAM mode-dispersion selectivity for classifying leads to the design of CNN, which can encode given images into various corresponding OAM states (Methods, Supplementary Fig. S4). Here, the learning task assigned to the CNN with a single convolutional layer and five diffractive layers is to encode the images of the handwritten digits [Modified National Institute of Standards and Technology (MNIST)] database into (LG_9,0_, LG_7,0_, LG_5, 0, …,_ LG_-9,0_) with an OAM order interval △*l* = 2. This tunable mapping relationship has been chosen to balance the high accuracy in encoding and the low crosstalk in decoding (Supplementary Fig. S5). As the beam size of the encoding modes doesn’t have obvious effects on the encoding accuracies (Supplementary Fig. S6), the encoding modes with same beam waists have been chosen in the CNN scheme throughout this manuscript. Notably, the weight coefficients of the OAM mode-dispersion impulse are trained directly in the CNN, which is significantly different from the previous free-space diffractive neural networks (DNNs) (Supplementary Note 6). In our architecture, the OAM mode-dispersion impulse components are determined by the OAM orders of the target LG modes. Moreover, to generate labeled LG modes with specific amplitude and phase distributions, a multi-task learning method is adopted in the design of loss function. After that, the CNN learns to distribute more power to the target encoding OAM states (Fig. 2c, Supplementary Fig. S7). To characterize the performance, the numerical testing encoding accuracy of the CNN can achieve 96.0% when trained by the MNIST test dataset (Fig. 2d). By further improving the classifier to 10 diffractive layers, the encoding accuracy could increase to 97.2% (Supplementary Fig. S8). Here, ten classes of images in the EMNIST dataset have been selected for mode-feature encoding, a similar encoding accuracy illustrates the robustness of the CNN (Supplementary Fig. S9). To illustrate the necessity of the OAM mode-dispersion impulse for mode-feature machine learning, the performances of the CNN and free-space DNN have been compared. To visualize the processes of these two neural network schemes, the similarities between ten randomly selected digital images in separate classes using OAM mode-features convoluted with/without OAM mode-dispersion impulse have been compared using the parameter of Euclidean distance (ED) (Materials and Methods). As can be seen, the convolutional operation with the trained OAM mode-dispersion impulse can increase EDs between the input OAM mode combs and decrease the similarities, resulting in an increase of the encoding accuracy from 70% to 96% (Supplementary Fig. S10).

### All-optical intelligent OAM-encoding of data-specific images for anti-eavesdropping wireless image transmission

For free-space communication using optical carrier to transfer information through an unguided channel^44^, OAM encoding enables not only high-density date transmission but also the improvement of security^45,46^, which is especially suitable for the atmospheric turbulence-free links ranging from inter-satellite or deep space mission to indoor directed wireless optical communications (WOCs)^47,48^. However, the current electronic computing for OAM encoding result in the challenges of the operation frequency gap and the computation efficiency of the whole information system. Here, OAM-mediated machine learning for all-optical information mode-feature encoding removes this hurdle. In the encoding part, four spatial light modulators (SLMs) were utilized to implement all-optical machine learning using a CNN comprising an input layer, a single convolutional layer and two diffractive layers, respectively. Three categories of Fashion MNIST database (T-shirt, Trouser and Ankle boot), 100 images in each category, were trained to be converted into LG_1,0_, LG_3,0_ and LG_5,0_, respectively (The amplitude/phase distributions of the convolutional layer and the classifier can be seen in the Supplementary Fig. S11). Limited by the phase/amplitude-only modulation capability, the amplitude part of the convolution layer, combined with the input image, was imprinted on the first SLM. After a propagation distance of ~3 m, the OAM information of the encoded beam was detected through adding diverse helical phase plates on the last SLM for inverse mode conversion.

**Fig. 3:**
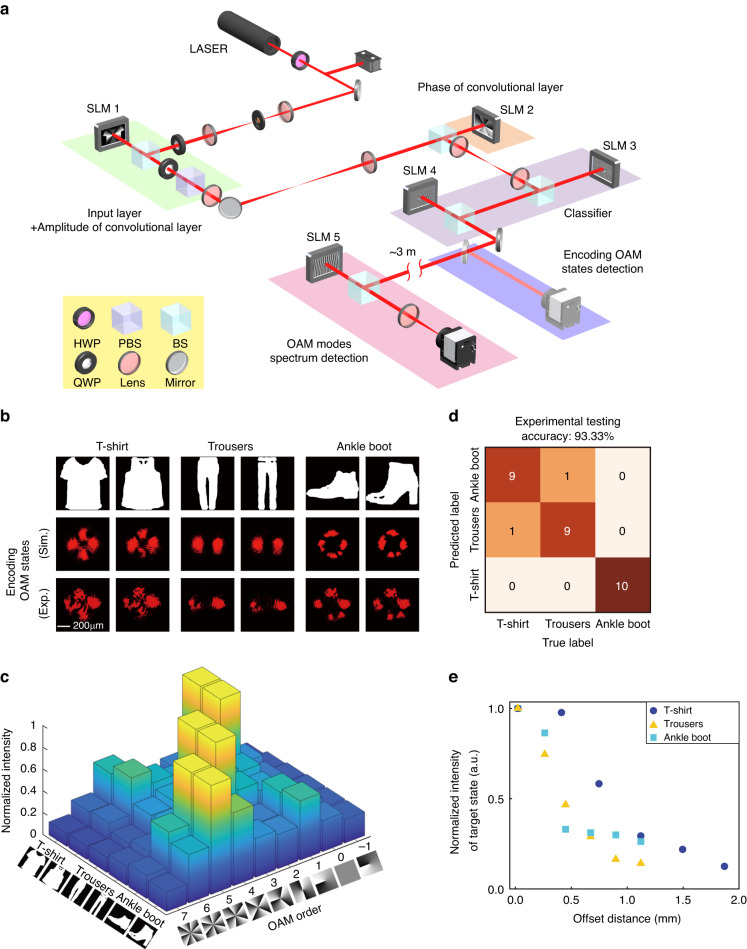
All-optical information mode-feature encoding in the application of anti-eavesdropping wireless image transmission. **a** The optical setup of the all-optical communication systems including encoding, transmission, and decoding. **b** The intensity distributions of the encoding OAM mode states for six testing images from the Fashion MNIST database. **c** The OAM information of the received beams. **d** The confusion matrix using 30 testing images and the overall testing encoding accuracy is 93.3%. **e** The influence of the deviation distance on the energy of target encoding OAM mode states

To be specific, as illustrated in Fig. 3a, a 632.8 nm beam from a He-Ne laser, passed through a half-wave plate (HWP) in combination with a polarization beam-splitter (PBS), which was used to continuously adjust the laser power. Through a beam expansion system consisting of 50-mm- and 400-mm- focal length- convex lenses, the light beam was illuminated on the first spatial light modulator (SLM, Hamamatsu, X13138). To achieve the amplitude-only modulation, two quarter wave plates (QWPs) were placed in the front and back of SLM 1. Through a 4-*f* system consisting of two convex lenses with 250-mm- focal length-, the electrical field including the amplitude part of the input image associated with the convolutional kernel was conjugated onto the SLM 2 (Holoeye, Pluto-2-NIR-011) where the phase part of the convolutional kernel was imprinted. After another 4-f system with an additional free-space propagation distance of 5 cm, the next two SLMs (SLM 3 and SLM 4, Hamamatsu, X13138) with a spacing distance of 15 cm were utilized to construct the classifier of the all-optical CNN. By inserting a folding mirror, the intensity of the encoding OAM modes at the output layer were separated and observed after a 15-cm propagation distance from SLM 4. In the application of WOC, the encoded OAM mode, after a ~3-m propagation distance, was illuminated on SLM 5 (Holoeye, GAEA-2-VIS-036). Finally, the OAM information was obtained by analyzing the intensity distributions collected in the CCD camera (Basler, acA2040-90uc).

For different input images, the experimental and theoretical results of the intensity distributions in the output layer are given in Fig. 3b. As can be seen from the OAM information detected after propagation in Fig. 3c, the designed CNN is capable of selectively distributing relative stronger energy into the target OAM modes corresponding to the input images. Here, the overall encoding accuracy was 93.3% using 30 images from the Fashion-MNIST test dataset (Fig. 3d). Notably, the lateral offset results in an inherent uncertainty in the measurement of OAM information. As such, the images encoded in this way are resistant to eavesdropping. To simulate the case that the eavesdropper could be compromised is the receiver located within a propagation divergence angle of ~0.00048° by alignment error, the OAM information was detected by adding a lateral offset on the last SLM for comparison. With an increasing deviation, the OAM components with the dominant energy differed from the target encoding OAM orders (Fig. 3e). As such, the encoded information was extremely difficult to infer, and the security of the WOC link was enhanced. Notably, a mode sorter can be applied in the detection of the OAM spectrum of the output beams, which can improve the detection efficiency (Supplementary Fig. S12)^29^. In addition, an OAM multiplexing hologram can be incorporated into the decoding part to achieve end-to-end switchable image display, which offers a pathway to realize all-optical information encoding, transmission, and display (Methods, Supplementary Fig. S13).

### All-optical dimension reduction for abnormal detection

**Fig. 4:**
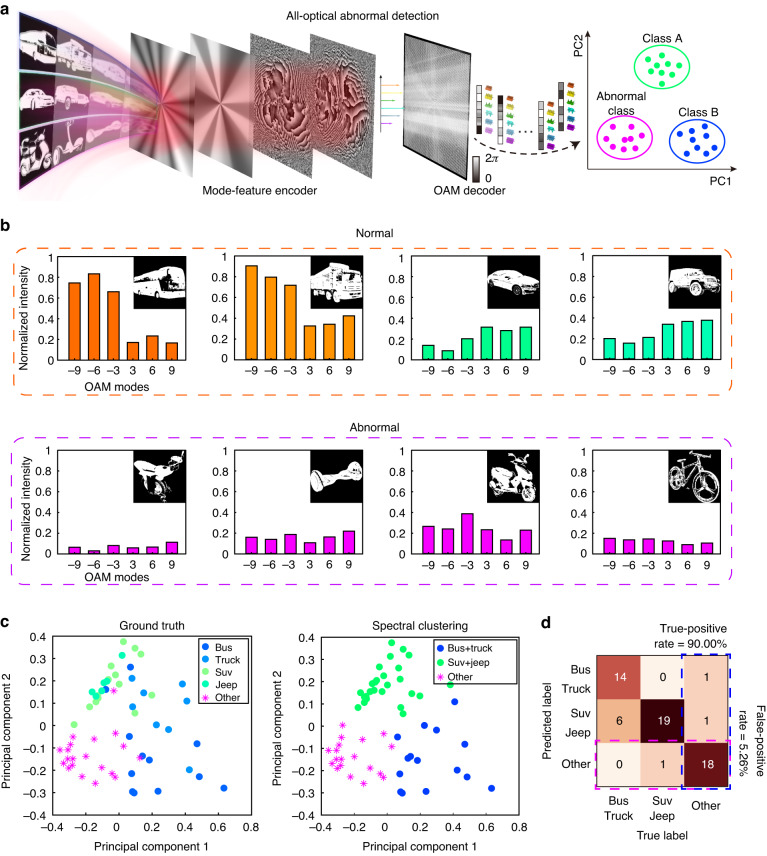
Information all-optical dimension reduction based on mode-feature encoded into six multiplexed OAM states. **a** The flowchart of all-optical dimension reduction-based abnormal detection, wherein CNN is utilized for OAM modes feature encoding and a fork-grating phase plate is introduced to achieve OAM modes decoding. **b** The decoded OAM information of the selected 4 normal images and 4 abnormal images. **c** The PCA and the spectral clustering results. **d** The confusion matrix illustrating the true-positive rate of classifying the abnormal image is 90.0% and the false-positive rate is 5.26%, respectively

More than a single OAM mode, introducing multiplexed OAM states as target encoding modes provides an unprecedented method for all-optical dimension reduction, which has significant impact on various further image sensing tasks, e.g. abnormal detection. Here, the CNN, with an output vector basis comprising six OAM states (*l* = −9,−6,−3,3,6,9), has been trained as an all-optical mode-feature encoder for two categories of car images (Fig. 4a). Therein, 80 “BUS” images have been encoded into (1,1,1,0,0,0) while 80 “SUV” images have been encoded into (0,0,0,1,1,1) in the training process, respectively. It is worthwhile mentioning that the weighting factors of several superposed LG modes with radial index *p* = 0 are optimized to obtain the target encoded mode basis (Supplementary Fig. S14). As an all-optical decoder to achieve the OAM information through a single measurement, we designed a fork grating containing six OAM states with the similar Fourier coefficients (Methods, Supplementary Fig. S15)^49^. After the decoder, the encoded image information after dimension reduction has been further applied in abnormal detection in our experiment. More than the previous two categories of images, the encoded mode-features of abnormal images have also been obtained through the all-optical CNN associated with the decoder. After principal component analysis (PCA) and spectral clustering (Supplementary Note 7), the accuracy of OAM-mediated machine learning for abnormal detection can be achieved.

As an example of optical inference, the intensity distributions after the decoder are given when four normal images and four abnormal images are selected as the input of the CNN for modes dimension reduction (Supplementary Fig. S16). After analyzing the intensities in each white dashed area representing each OAM basis, the images with a resolution of 256*256 can be compressed into 1*6 matrix (Fig. 4b). Then, a PCA was performed on all these 6-dimensional feature vectors, wherein the first two principal components were normalized to a similar scale and illustrated in the left panel of Fig. 4c. Notably, the data points corresponding to the anomalous car images can be separated from the other categories of car images, which provides the ground truth labels for calculating the percentage of recognizing anomalous images as well as classifying the normal images. Through comparing the data after spectral clustering algorithms, the confusion matrix can be obtained with an overall accuracy of 85%, the true-positive rate of classifying the abnormal image is 90.0% and the false-positive rate is 5.26%, respectively (Fig. 4d).

## Discussion

The OAM-mediated machine learning with all-optical CNNs provides a universal mechanism to transform the data feature into the OAM states, which can remove the hurdle of optically OAM information encoding towards higher-throughput and lower latency image-sensing applications. In the specific application of wireless image transmission, the proposed CNN with a high encoding accuracy opens the door to encode the specific databases and images at the speed of light, leading to an all-optical anti-eavesdropping WOC system due to the inherent ultrasensitive measurement requirements of OAM information. Furthermore, the employment of multiplexing mode states as target encoding states of the CNN can break the bottleneck of optically dimension reductions in the OAM domain, reaching a compressing ration of ~10^4^ in comparison to the input data in the space domain.

Along with a phase-only OAM mode-dispersion impulse^50^, the phase-only CNNs for OAM-mediated machine learning can be achieved. To reduce the expenses of the system, the SLMs can be replaced by the passive optical elements, such as the compact planar optical elements based on metasurface holograms^51,52^ and patterned liquid crystals holograms^53,54^. To achieve the high degree of the device integration, one can adopt nanophotonic and optoelectronic devices with high spatial resolution and physical connectivity to construct the on-chip CNN with a high neuron density^55–57^. In terms of the parallelism, the large-area diffractive devices are also desired to achieve high-efficient all-optical machine learning-based information mode-feature encoding^58^. In addition to encoding into the OAM dimension of LG modes, the CNN proposed here can also be extended to encoding the LG modes with the distinctive radial index, which can further increase the capacity of optical systems^59^. It can be expected that with experimental realizations of free-space ONN-based OAM sensing technologies^60^, the proposed CNN associated with representation learning can facilitate various applications including all-optical machine learning-based high-capacity holographic communications, LIFI, cellular deformation classification, and face similarity recognition.

## Materials and methods

### Forward propagation model and error backpropagation of the CNN

#### Forward propagation model

The forward model of our CNN architecture in the TensorFlow implementation is summarized in Supplementary Fig. S4. The input mode at layer 0 ${h}_{i}^{0}$ is usually a complex-amplitude value, which can carry information in its phase and/or amplitude channel. After the convolutional layer, the wave function ${u}_{i}^{0}$ generated by the interaction between the input light field ${h}_{i}^{0}$ and the OAM mode-dispersion impulse ${c}_{i}^{0}$ can be written as

1 $$\left\{\begin{array}{l}{h}_{i}^{0}={\sum }_{l=-\infty }^{+\infty }{\tilde{A}}_{l}\exp ({\rm{j}}l\phi )\\ {c}_{i}^{0}={\sum }_{l=-\infty }^{+\infty }{\tilde{B}}_{l}\exp ({\rm{j}}l\phi )\\ {u}_{i}^{0}={h}_{i}^{0}{c}_{i}^{0}\end{array}\right.$$

where $\widetilde{{A}_{l}}$ and $\widetilde{{B}_{l}}$ represent the complex-amplitude weighting coefficients of the OAM mode spectrums, respectively, and *i* represents the *i*-th neuron located at (*x*_*i*_, *y*_*i*_).

Following the angular spectrum diffraction equation, the complex-amplitude field of each neuron passing through the classifier of the given CNN can be expressed as

2 $${u}_{i}^{n+1}(x,y)={\Im }^{-1}\{\Im \{{u}_{i}^{n}({x}_{i},{y}_{i}){t}_{i}^{n}({x}_{i},{y}_{i})\}H({f}_{x},{f}_{y})\}$$

where *n* represents the *n*-th layer of the classifier of the CNN. Moreover, $\Im$ represents the Fourier transform, and ${\Im }^{-1}$ represents the inverse Fourier transform. The transmission coefficient of a neuron is composed of amplitude and phase terms, i.e., ${t}_{i}^{n}({x}_{i},{y}_{i})={A}_{i}^{n}({x}_{i},{y}_{i})\exp (j{\varphi }_{i}^{n}(x_{i},{y}_{i}))$. For a phase-only CNN architecture in our experiment, the amplitude ${A}_{i}^{n}({x}_{i},{y}_{i})$ is assumed to be a constant, ideally 1. $H({f}_{x},{f}_{y})$ is the transfer function in the spatial frequency domain and the phase delay factor related to propagation distance *d*, i.e. ${\exp}(\left(\right.\!{\scriptstyle{j2{\pi} d} \atop }/{\lambda})\sqrt{1-{(\lambda {f}_{x})}^{2}-{(\lambda {f}_{y})}^{2}}$, *λ* is the illumination wavelength, $j=\sqrt{-1}$. Accordingly, the forward propagation model can be described. If the CNN design is composed of M layers (excluding the input and output planes), a complex field can be obtained in the output plane, which is expressed as

3 $${u}_{i}^{M+1}={a}_{i}^{M+1}+{b}_{i}^{M+1}j$$

where ${a}_{i}^{M+1}$ and ${b}_{i}^{M+1}$ represent the real part and imaginary part of the output light field, respectively. When compared with the target label, the error is propagated back to update the layer of CNN iteratively, which is described in detail below.

#### Error backpropagation

To train the CNN, the error back propagation algorithm and the random gradient descent optimization method are implemented on TensorFlow framework. A loss function is defined to evaluate the inconsistency between the target real value ${\bar{u}}_{i}^{M+1}$ and the predicted value of the network output, which is termed as the mean square error (MSE). And the target real value can be written as:

4 $${\bar{u}}_{i}^{M+1}={\bar{a}}_{i}^{M+1}+{\bar{b}}_{i}^{M+1}j={\sum }_{l=-\infty }^{+\infty }{C}_{l}\exp ({\rm{j}}l\phi )$$

where ${C}_{l}$ represents the amplitude-only coefficients (without phase differences) of the OAM mode comb. ${\bar{a}}_{k}^{M+1}$ and ${\bar{b}}_{k}^{M+1}$ represent the real part and imaginary part of the target light field, respectively. Specifically, the loss function of the CNN is given as

5 $$Loss=\alpha \frac{1}{k}{\sum }_{k}{\left({a}_{k}^{M+1}-{\bar{a}}_{k}^{M+1}\right)}^{2}+\beta \frac{1}{k}{\sum }_{k}{\left({b}_{k}^{M+1}-{\bar{b}}_{k}^{M+1}\right)}^{2}$$

where *k* refers to the number of measurement points at the output plane. The parameters (*α* and *β*) represent the adjustment coefficients of the two loss functions, respectively (Throughout this manuscript, *α* = *β* = *0.5*). To meet the objective requirements, the relevant parameters are optimized through continuous iterations of the neural network to minimize the loss function.

Specifically, our CNN architectures were trained using Python (v3.9.7) and TensorFlow (v2.6.0, Google Inc.) on a server with a NVIDIA Quadro P4000 graphical processing unit (GPU), and Intel(R) Xeon(R) W-2133 central processing unit (CPU, Intel Inc.) and RAM of 32 GB. The batch size and learning rate were set as 100 and 0.03, respectively. The resolution (neuron number) of each layer of the classifier was 256*256, 256*256, and 400*400 for Figs. 2–4, respectively. Finally, the complex-amplitude of the convolutional layer and the phase distributions of the classifier were achieved after 2000, 4000 and 4000 epochs for Figs. 2–4, respectively.

### Calculation of Euclidean distance using OAM mode basis

Usually, the Euclidean distance (ED) is a measurement of the straight-line distance between two points in Euclidean space. In two-dimensional space, the ED between two points can be calculated by the length of the line segment connecting the two points. In higher-dimensional spaces, the calculation is similar, but involves more coordinates. Here, the information has been expressed into OAM basis to achieve OAM-mediated machine learning. As such, the ED of two matrix-vectors *X*_*k*_ and *Y*_*k*_ using OAM mode-features can be expressed as

6 $$ED=\sqrt{\mathop{\sum }\limits_{k=1}^{Length}{({X}_{k}-{Y}_{k})}^{2}}$$

where the vector elements of the *X*_*k*_ and *Y*_*k*_ are represented by the energy (square of the amplitude) weighting coefficients of the OAM mode components constituting the electrical fields.

### Design of the OAM multiplexing hologram for end-to-end switchable image display

The conceptual illustration of OAM-mediated machine learning-based end-to-end switchable image display is shown in Supplementary Fig. S13a. In comparison to the application in Fig. 3, the significant difference is the information can be displayed at the receiver. As such, the decoding process using the electrical circuits is further replaced.

And the optical setup in Fig. 3a was utilized to demonstrate this concept. The CNN was designed to encode two handwritten “digit” images (“1” and “2”) into the LG_4,0_ and LG_-4,0_, respectively. After a 3-meter propagation distance, the encoding beam illuminated on the OAM multiplexing hologram which was imprinted on the last SLM. The design of the OAM multiplexing hologram is shown in Supplementary Fig. S13b. Firstly, the phase-only OAM preserved holograms of these two handwritten “digit” images are designed through an iterative Fourier transformation algorithm. After complex superposing these two OAM-preserved holograms encoded with helical phases distributions with *l* = 4 and −4, the final phase term was used as OAM-multiplexing hologram. In the experiment, when a handwritten digit image (as well as the amplitude of the convolutional kernel) was imprinted on the first SLM, the images were observed on the CCD camera (Supplementary Fig. S13c). Notably, the task of switchable image display, including encoding, transmission, and display, was performed at speed of light.

### Design of fork gratings for detecting OAM information of encoding light beams through a single measurement

To decode the OAM information of encoding light beams through a single measurement, a fork grating function was designed to convert OAM components into Gaussian modes at different positions. The transmission function of the fork grating *f(φ)* can be expressed as

7 $$f(\varphi )=\sum _{m}{A}_{{l}_{m}}\exp (i{l}_{m}\varphi )\exp (i{g}_{m})$$

where *l*_*m*_ and *A*_*lm*_ denote the OAM order and the amplitude weighting coefficient of the elementary grating function *g*_*m*_, respectively. Obviously, *f*(*φ*) appears in a complex-amplitude form normally.

To achieve an approximate form for the phase-only grating function *g(φ)*, which can be defined as

8 $$g(\varphi )=\exp [ip(\varphi )]$$

where *p(φ)* can be expressed as

9 $$p(\varphi )=\mathrm{Re}\left\{-i\,\mathrm{ln}\left[\mathop{\sum }\limits_{m=1}^{N}{B}_{{l}_{m}}\exp (i{l}_{m}\varphi )\exp (i{g}_{m})\right]\right\}$$

where *B*_*lm*_ is a decisive factor for *p(φ)*, Re{} denotes the symbol of “real part”, Expanding *g(φ)* into the Fourier series given by

10 $$g(\varphi )=\mathop{\sum }\limits_{m=-\infty }^{\infty }{C}_{{l}_{m}}\exp (i{l}_{m}\varphi )\exp (i{g}_{m})$$

where the decomposition coefficient *C*_*lm*_ can be expressed as

11 $${C}_{{l}_{m}}=\frac{1}{2\pi }{\int }_{\!0}^{2\pi }g(\varphi )\exp (-i{l}_{m}\varphi )\exp (-i{g}_{m})d\varphi$$

Limited by the phase-only SLM in our experiment, the optimizing algorithm introduced below is adopted to ensure the phase-only transmission function *g(φ)* and the original function *f(φ)* are approximately consistent. Firstly, *η*_*m*_, the diffraction efficiency of the grating at the mth-order, is defined as

12 $${\eta }_{m}=\frac{{C}_{{l}_{m}}}{{C}_{l}}$$

where ${C}_{l}=(1/2\pi ){\int }_{0}^{2\pi }g(\varphi )\exp (-i{l}_{m}\varphi )d\varphi$ represents the total energy.

Afterwards, another parameter *U* is adopted to evaluate the diffraction efficiency and uniformity among different diffraction orders simultaneously,

13 $$U=1-\frac{{\eta }_{{\rm{m}}}(\max )-{\eta }_{{\rm{m}}}(\min )}{{\eta }_{{\rm{m}}}(\max )+{\eta }_{{\rm{m}}}(\min )}$$

Here, *η*_*m*_ (max) and *η*_*m*_ (min) represent the maximum and minimum values of *η*_*m*_, respectively.

The flow chart to obtain the phase-only fork grating function is shown in the figure below.
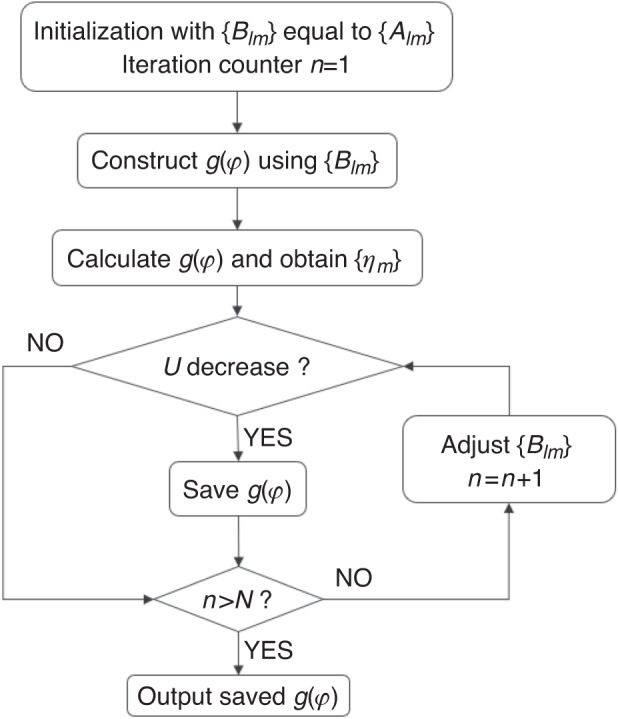


As an initiation of the process, we set *B*_*lm*_ *=* *A*_*lm*_ and the iteration counter *n* = 0. According to Eqs. (9)–(11), *η*_*m*_ and U can be calculated. When the iteration counter n is less than the pre-set number N, the phase-only grating *g(φ)* is optimized through continuously updating the *B*_*lm*_.

14 $$\begin{array}{c}|{B}_{{l}_{m}}^{\text{'}}|=|{B}_{{l}_{m}}|+\beta (|{A}_{{l}_{m}}|-|{C}_{{l}_{m}}|)\\ {B}_{{l}_{m}}^{\text{'}}=\frac{|{B}_{{l}_{m}}^{\text{'}}|}{|{C}_{{l}_{m}}|}\ast {C}_{{l}_{m}}\end{array}$$

Here, || denotes the symbol of the “amplitude part”. And *β* is a constant representing the update rate. Finally, a phase-only fork grating function featuring different OAM orders in different diffraction orders with high efficiency and high uniformity can be obtained. The performance is shown is shown in Supplementary Fig. S15. When the fork-grating in Supplementary Fig. S15a was illuminated by the Gaussian beam, the intensity distribution is shown in Supplementary Fig. S15b. To obtain the OAM order and intensity of each spot, the fork-grating was illuminated by the OAM beams with *l* ranging from −9 to 9 with an interval of 3. Due to the OAM mode conservation, the Gaussian spot appears in different positions (Supplementary Fig. S15c). As can be seen from Supplementary Fig. S15d, the uniformity of this fork grating is 0.942.

### Supplementary information

Supplementary Information for Orbital angular momentum-mediated machine learning for high-accuracy mode-feature encoding
