# Supplementary material for: Orbital angular momentum-mediated machine learning for high-accuracy mode-feature encoding
Source: Light Sci Appl. 2024 Feb 14;13:49. doi: 10.1038/s41377-024-01386-5 (PMC11251042; doi:10.1038/s41377-024-01386-5)
Supplement: Supplementary file 1 — Supplementary Information for Orbital angular momentum-mediated machine learning for high-accuracy mode-feature encoding [file 41377_2024_1386_MOESM1_ESM.docx]

Supplementary Information for

**Orbital angular momentum-mediated machine learning for high-accuracy mode-feature encoding**

Xinyuan Fang^1,3, *^, Xiaonan Hu ^1,2,3^, Baoli Li ^1,3^, Hang Su^1,2^, Ke Cheng^1,2^, Haitao Luan ^1^, and Min Gu^1, *^

^1^ Institute of Photonic Chips, University of Shanghai for Science and Technology; Shanghai, 200093, China.
^2^ Centre for Artificial-Intelligence Nanophotonics, School of Optical-Electrical and Computer Engineering, University of Shanghai for Science and Technology; Shanghai, 200093, China.

^3^ These authors contributed equally: Xinyuan Fang, Xiaonan Hu, Baoli Li.
^*^Corresponding author. Email: [xinyuan.fang@usst.edu.cn](mailto:xinyuan.fang@usst.edu.cn); [gumin@usst.edu.cn](mailto:gumin@usst.edu.cn).

**This file includes:**

Supplementary Notes 1 to 7

Supplementary Figures 1 to 16

Supplementary Text

**Supplementary Note 1: Convolution of an OAM mode comb with an OAM mode-dispersion impulse based on superposed electrical fields in the spatial domain.**

The superposition of electrical fields in the spatial domain results in the convolution of an OAM mode comb with an OAM mode-dispersion impulse (Fig. 2A). The detailed mathematic proof is given below.

The images *U* can be decomposed into a series of coherent OAM modes, which is termed as the OAM mode comb given by

 (S1)

where *g*(*l_x_*) represents the complex-amplitude weighting coefficients of the OAM order *l_x_* in the OAM mode comb. Different from the spatial frequency spectrum, a Fourier lens is not required to achieve the OAM mode spectrum.

Similarly, the OAM mode-dispersion impulse of a complex-amplitude field *E* can be expressed as

 (S2)

where *h*(*l_y_*) represents the complex-amplitude weighting coefficients of the OAM order *l_y_* in the OAM mode-dispersion impulse. Based on the OAM conservation law, a superposition of the image and the complex-amplitude field results in the broadened OAM mode comb *f*(*n*), which can be expressed as

$f\text{(}n\text{)=}\sum_{\tau=-\infty}^{+\infty} \text{g}(\tau)h(n-\tau)\text{=(g}\otimes h)(n)$ (S3)

where *f*(*n*), *g*(*τ*) and *h*(*n*-*τ*) represent the weighting coefficients of the OAM order *l*= *n*, *τ,* *n*-*τ*, respectively.

As a result, the superposed electrical fields in the spatial domain can be mathematically defined as a one-dimensional convolution operation for an OAM mode comb with the OAM mode-dispersion impulse, wherein the OAM mode-dispersion impulse *h*(*l_y_*) is termed as the convolution kernel in a neural network.

**Supplementary Note 2: The diffraction losses of distinctive Laguerre-Gaussian (LG) modes in a single** **diffractive layer with finite size.**

To achieve OAM mode dispersion-selectivity, the diffraction losses of distinctive LG modes are analyzed to illustrate the capability to control the evolution of the OAM mode combs.


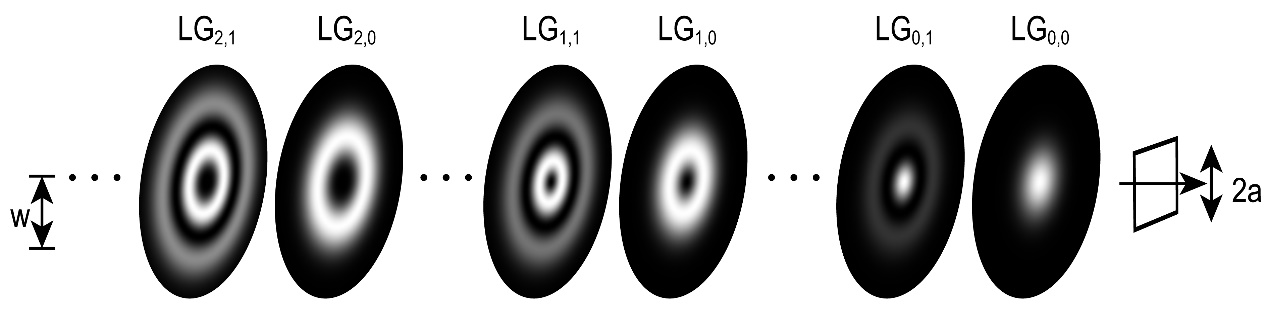


Without losing generality, the model of a single diffractive layer is adopted as shown in the figure above. In the cylindrical coordinate (*r*, *φ*, z), the complex field of a LG mode at the waist plane (z=0) can be described as

 (S4)

where $\text{L}_{p}^{\left|l\right|}$ is the generalized Laguerre polynomial, *w* represents the beam waist, *l* is the OAM order (the topological charge), and *p* is the radial index of a LG mode, respectively. And the intensity distribution *I_LG_* can be expressed as

 (S5)

where *C* is a constant. When LG_l,p_ beam mode passes through a diffractive layer with finite size which can denoted by *S*, the transmittance *t_l,p_* of the module of the amplitude can be expressed as

 (S6)

Here, the diffraction loss δ*_l,p_* can be defined as

$\delta_{l,p}=1-t_{l,p}$ (S7)

**Supplementary Note 3: Evolution of the Laguerre-Gaussian (LG) mode basis after the diffractive layer with finite size.**


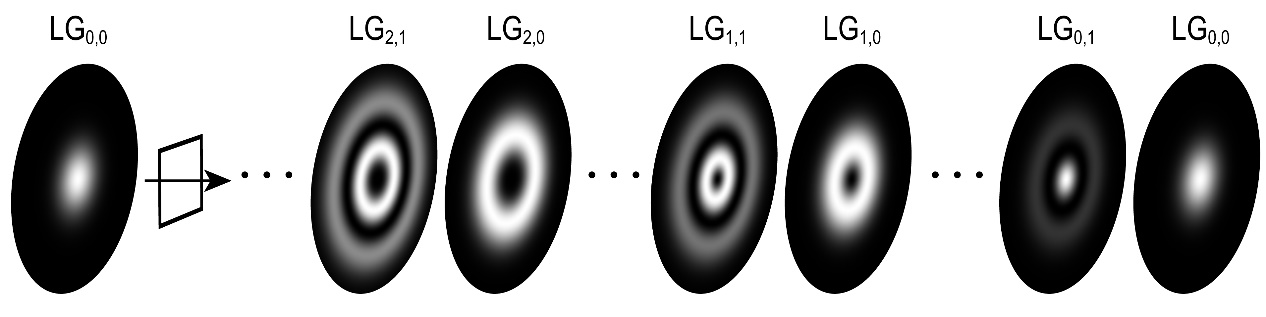


Except for the loss of the total energy (or the module of the amplitude) due to the diffractive layer with finite size, a specific LG mode is converted into a LG mode combination with complex-amplitude weight coefficients. When the LG beam mode passes through the diffractive layer with finite size described by the transmission function *E_T_*, a complex-amplitude LG mode combination *A_T_(l,p)* can be achieved based on

 (S8)

To analyze the evolution of the LG mode basis of an arbitrary electrical field due to the diffractive layer with finite size, the broadened LG mode combinations *A_T_*(*l*,*p*) should be multiplied by the transmittance *t_l,p_* of a specific LG mode, which can be seen as a weighting factor (Fig. S2).

**Supplementary Note 4: Evolution of the OAM mode comb after the diffractive layer with finite size.**


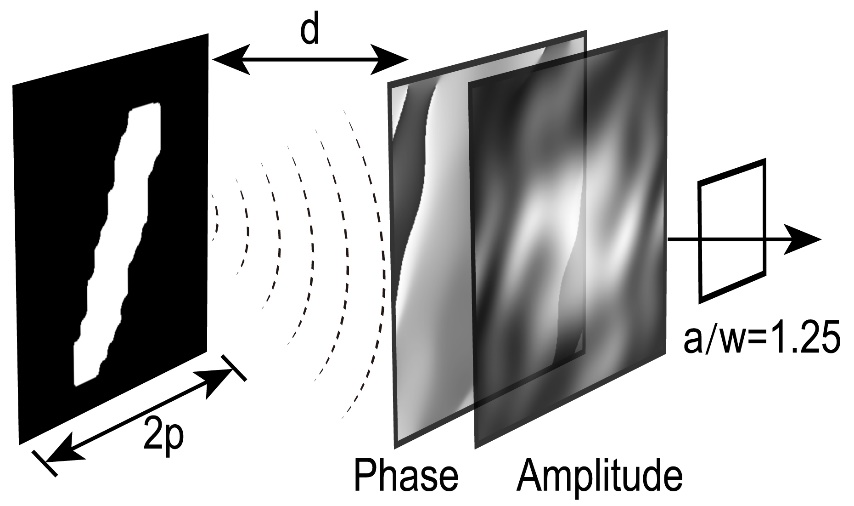


As an example, the diffraction loss of distinctive LG modes is implemented to analyze the evolution of the OAM mode comb of the handwrite digit “1”. Firstly, the electrical field of a handwrite digit “1” *U*_hw_ after a propagation distance of *d=100λ* can be expressed as

$U_{\text{hw}}\left(x,y\right)=\frac{\exp\left(ikd\right)}{i\lambda d}\int\int_{-p}^{p} u_{hw}\left(x_{1},y_{1}\right)\exp\left\{\frac{i\pi}{\lambda d}\left[\left(x-x_{1}\right)^{2}+\left(y-y_{1}\right)^{2}\right]\right\}dx_{1}dy_{1}$ (S9)

Here, *x_1_-y_1_* and *x-y* are the coordinates in the image plane and the aperture plane, respectively. 2*p=25λ* is the width of the handwrite digit image, *λ* is the wavelength, and *k* represents the wave vector. The electrical field *U*_hw_ can be decomposed into an OAM mode comb *u*_hw_(*l*), which is expressed as

 (S10)

Furthermore, it can be decomposed into the complex-amplitude LG mode spectrum *A*_hw_(*l,p*), which can be expressed as

 (S11)

According to the evolution of the various LG mode basis which is introduced in Supplementary Note 4, the updated OAM modes spectrum can be obtained.

**Supplementary Note 5: Cascaded diffractive layers with OAM mode-dispersion selectivity for pose recognition.**

To experimentally demonstrate that distinctive diffractive losses of LG modes and OAM mode conversion results in the OAM mode-dispersion selectivity, we implemented cascaded diffractive layers for machine learning to encode a specific category of images with various squatting poses into the LG_4,0_ mode and applied it in the pose recognition (Fig. S3a).

Firstly, 10 images of various squatting poses were captured and pre-processed into binary amplitude distribution. Here, we consider coherent illumination with a wavelength of 632.8 nm, and the free-space propagation module is implemented using the angular spectrum method. The hidden layers can be experimentally created by using two cascaded reflective spatial light modulator (SLMs 2 and 3), wherein the phase modulation coefficient at each neuron can be iteratively adjusted during the training using error back-propagation method. It is worthwhile mentioning that to generate a specific LG_4,0_ mode, the loss function should include the amplitude term and the phase term. After this numerical training process with 8 images through a computer after 2000 epochs, the phase level of the neurons embedded in SLMs is determined.

To perform the task at the speed of light, the optical setup is illustrated in Fig. S3b. Following the design, the axial distance between the input layer, two hidden layers and output layers are 5 cm, 15 cm, and 15 cm, respectively. And the OAM modes spectrum were achieved at the output plane by imprinting distinctive helical phases on the last SLM. For all the 10 input images, the designed two cascaded diffractive layers can distribute most energy into the appropriate OAM channel with *l*=4 (Figs. S3c and d).

As introduced in the main text, the OAM mode-dispersion selectivity can be attributed into two reasons: the diffraction loss and OAM modes conversion. To prove our analysis, we individually analyze the phase distribution of the second layer by illuminating a Gaussian beam. As can be seen in Fig. S3e, the dominant energy is distributed in the OAM channel with *l*=4. As a result, this proves that before the second layer, the fundamental Gaussian mode is selected from the LG modes spectrum of the input images due to the relatively low diffractive loss.

**Supplementary Note 6: Comparisons of the CNN with previous DNN in the forward propagation model and error backpropagation.**

The differences in this aspect between the CNN and the DNN can be summarized below.

Firstly, in the CNN framework, the OAM mode-dispersion impulse is a superposed OAM state, wherein the weight coefficients of each OAM component is a trainable real number. Secondly, the constraint mode of phase variable layer in CNN is set to the constraint of exponential taking angle. The sigmoid function in DNN only maps a real number to the interval of (0,1). When the input value approaches positive infinity or negative infinity, the gradient will be close to zero, thus causing the gradient dispersion. If combined with the gradient descent method, the weights of the initial several layers will change very slowly and cannot be effectively trained. On the contrary, the constraint of exponential taking angle may prevent this problem. As such, in our case for the training of complex-amplitude electrical field, it is required to constrain the phase variable, which can directly map a real number to the interval of (-π, π). Furthermore, the loss function in DNN is the MSE of the intensity between the output light field and the target light field. In comparison, the task of encoding on the OAM dimension of complex-amplitude LG modes illustrates the loss function should be divided into two parts, including the MSE of the real part and the imaginary part of the electrical field.

**Supplementary Note 7: The detailed descriptions of the principal component analysis algorithm and the spectral clustering algorithm.**

Principal component analysis (PCA) is an algorithm for achieving dimension reduction. In our experiment, after collecting the 1*6-dimension matrix-vectors in OAM basis of the 60 selected test samples, the covariance matrix has been computed to obtain the matrix's eigenvalues and corresponding eigenvectors. Next, the largest 2 eigenvectors corresponding to the largest eigenvalues are selected as the principal components. Then, through calculating the inner product of the sample data and the eigenvector, the scores of each data point on each principal component can be obtained, which indicates the projection of the sample data onto the selected principal components. Finally, the visualization data shown in Fig. 4c (left panel) is produced using the scores obtained from the two sets of principal components.

Spectral clustering, derived from graph theory, is a standard statistical algorithm which can reveal the similarities. It mainly can be divided into 7 steps: 1) similarity computation; 2) construction of the similarity matrix; 3) normalization of the similarity matrix; 4) construction of the Laplacian matrix; 5) eigenvalue decomposition; 6) dimensionality reduction; 7) clustering. Here, a standard spectral clustering algorithm has been utilized to process the data after PCA dimensionality reduction in Fig. 4c (left panel), which can be viewed as points in a two-dimensional space. Notably, edges can be used to connect the points. Specifically, for the given two data points *x_i_* and *x_j_*, the edge weights can be obtained by computing the Gaussian kernel function$exp(-\left\| x_{i}-x_{j} \right\|^{2}/(2\sigma^{2}))$. Here, σ is the bandwidth parameter of the Gaussian kernel function, which is used to control the decay rate of similarity. And represents the norm of a vector, which can be used to calculate the Euclidean distance between vectors. As such, the edge weight between two distant points is low, while the edge weight between two nearby points is high. Through minimizing the sum of edge weights between different subclass and maximizing the sum of edge weights within each subclass, we can finally achieve clustering as shown in Fig. 4c (right panel).


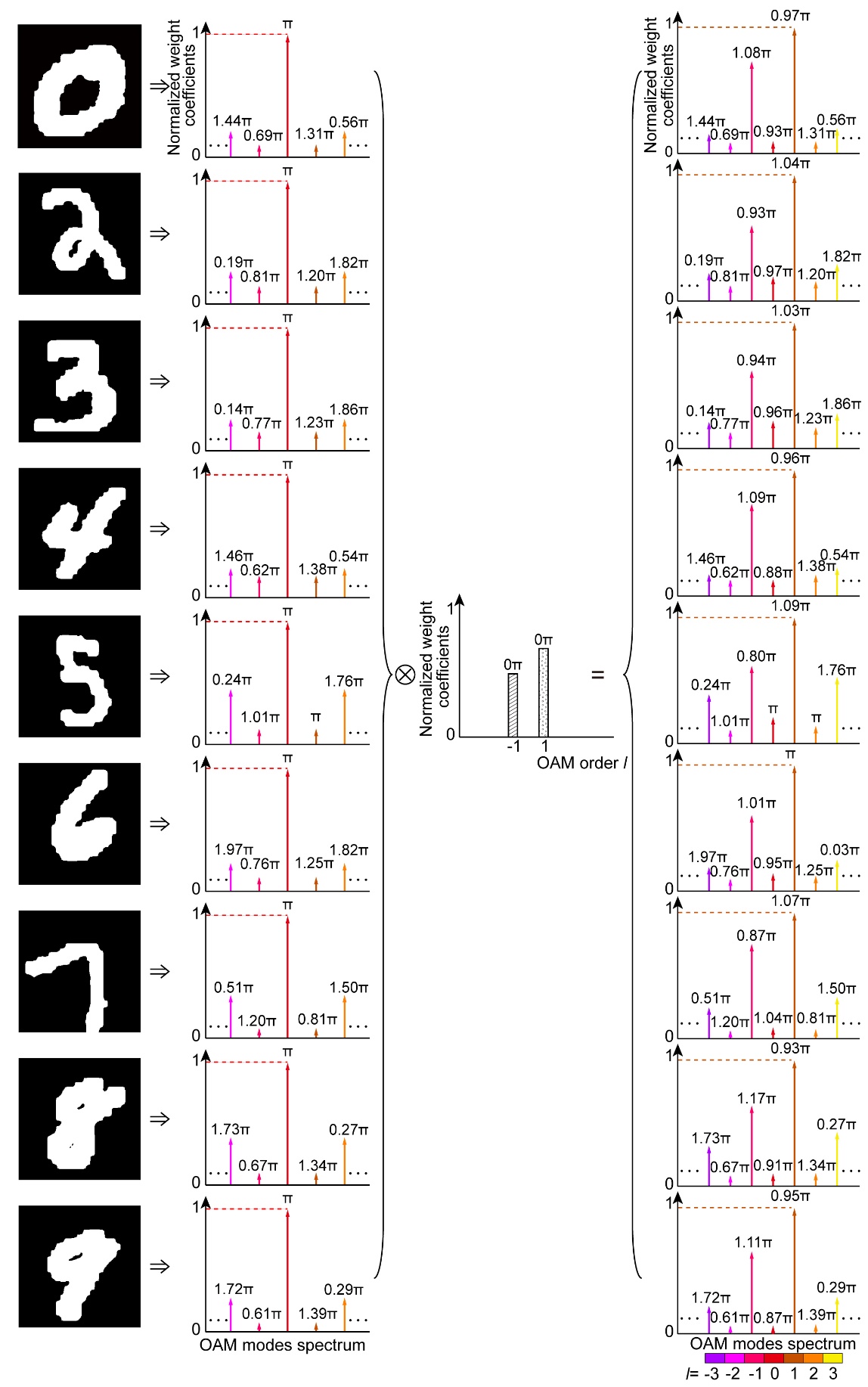


Fig. S1. Convolution of the OAM mode combs of the handwrite digits “0”, and “2” to “9” with the OAM mode-dispersion impulse in Fig. 2a.


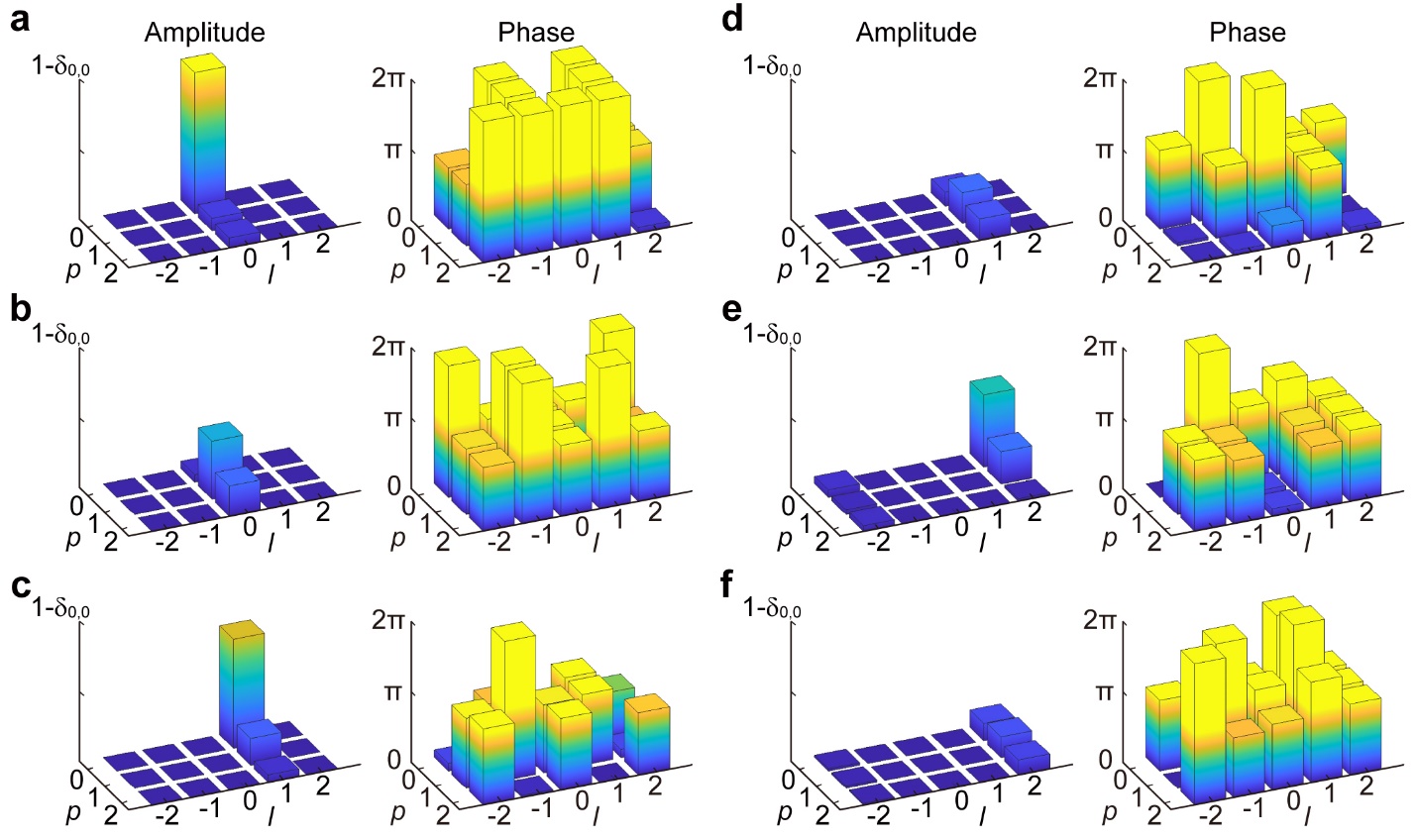


**Fig. S2. Evolution of the selected LG mode basis ((a)-(f) LG_0,0_, LG_0,1_, LG_1,0_, LG_1,1_, LG_2,0_, LG_2,1_) after the diffractive layer with a scaling factor a/w of 1.25.**

**
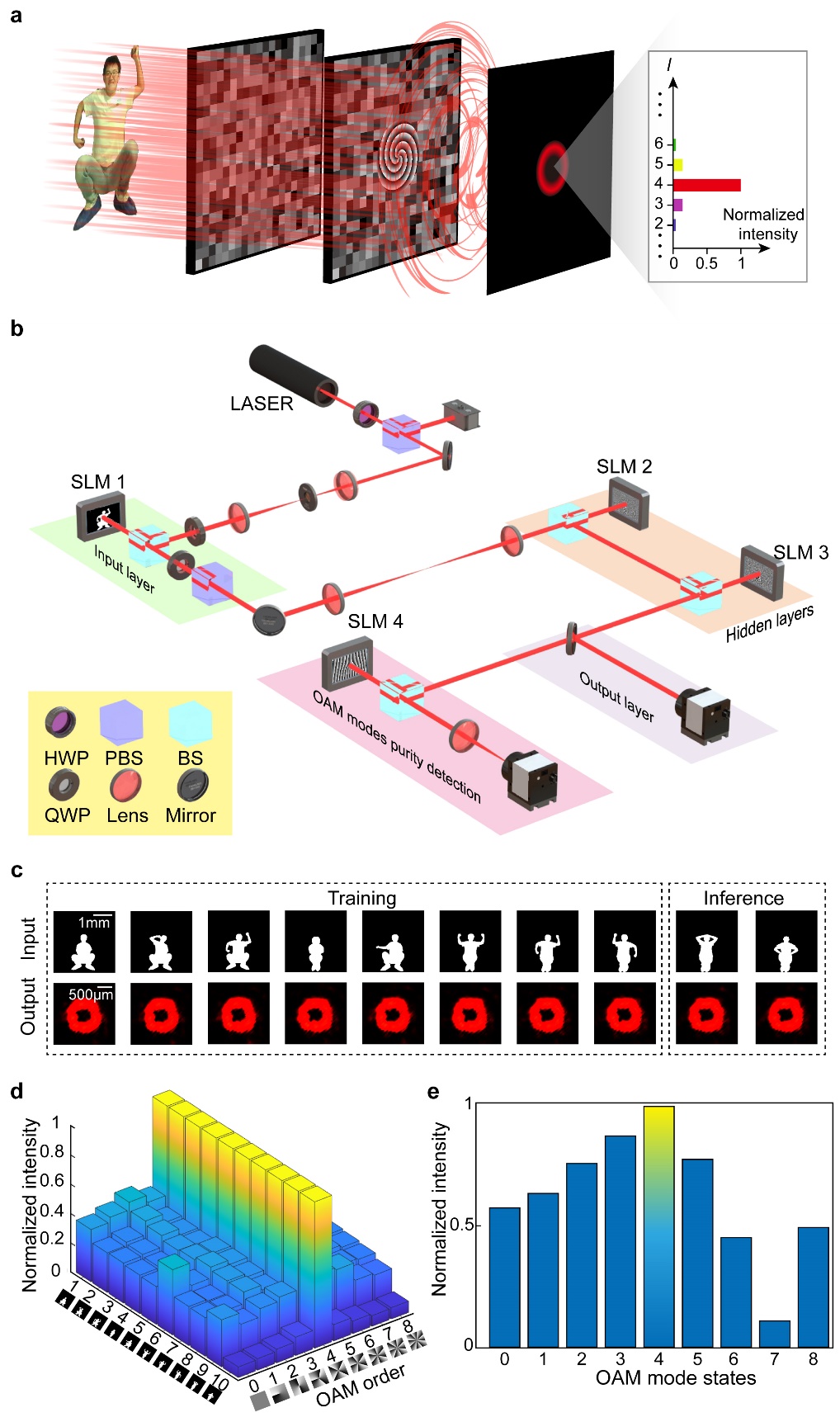
**

**Fig. S3.** **Experimental demonstration of OAM mode-dispersion selectivity for pose recognition.** **a,** Conceptual illustration of cascaded diffractive layers for OAM-based pose recognition. **b,** Optical setup. **c,** Experimental results of the encoding OAM states. **d,** Experimental results of the OAM modes spectrums. **e,** OAM mode spectrum at the output plane when the second layer illuminated by the Gaussian beam.


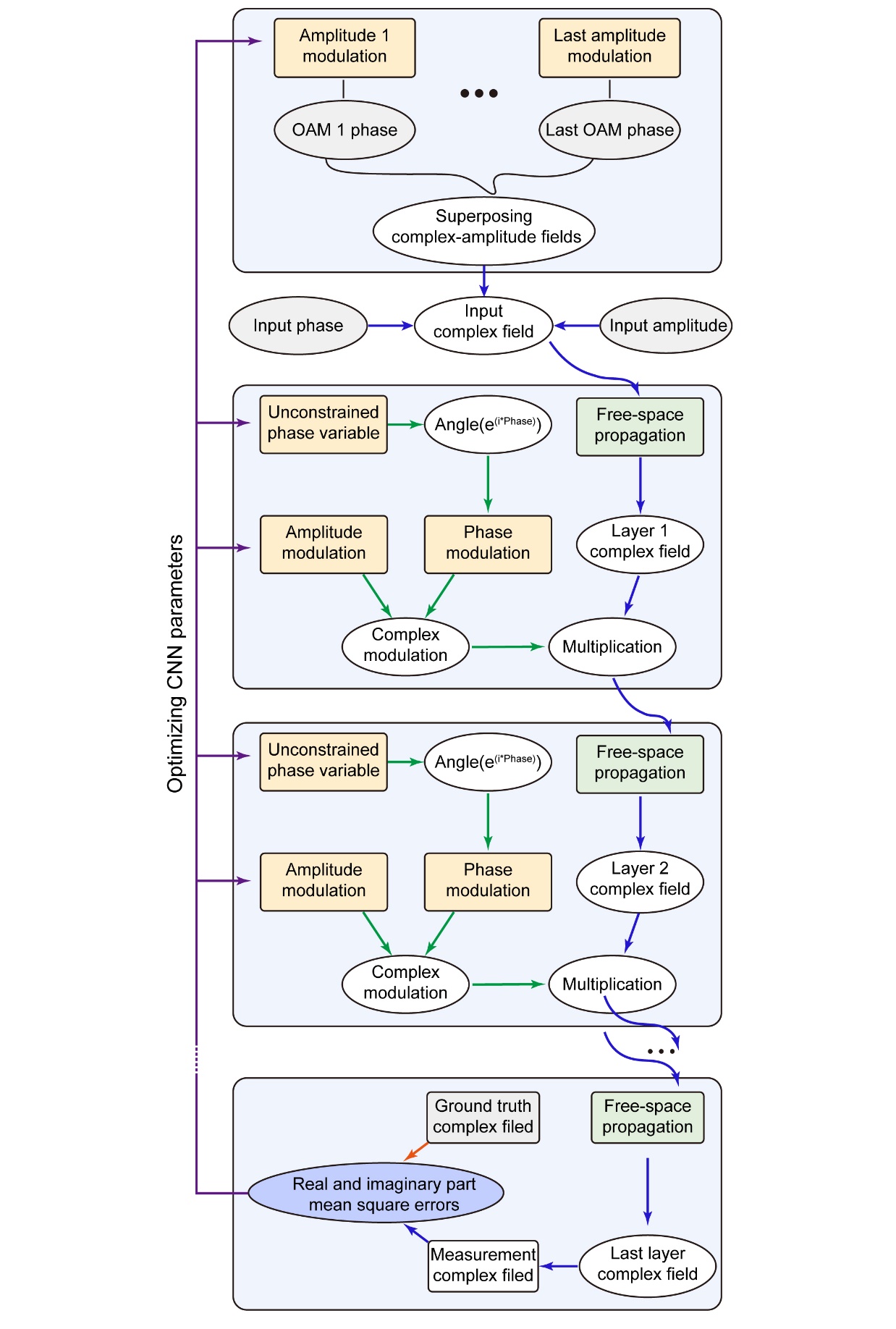


**Fig. S4. Flowchart of the forward propagation model and error backpropagation method of the CNN.**


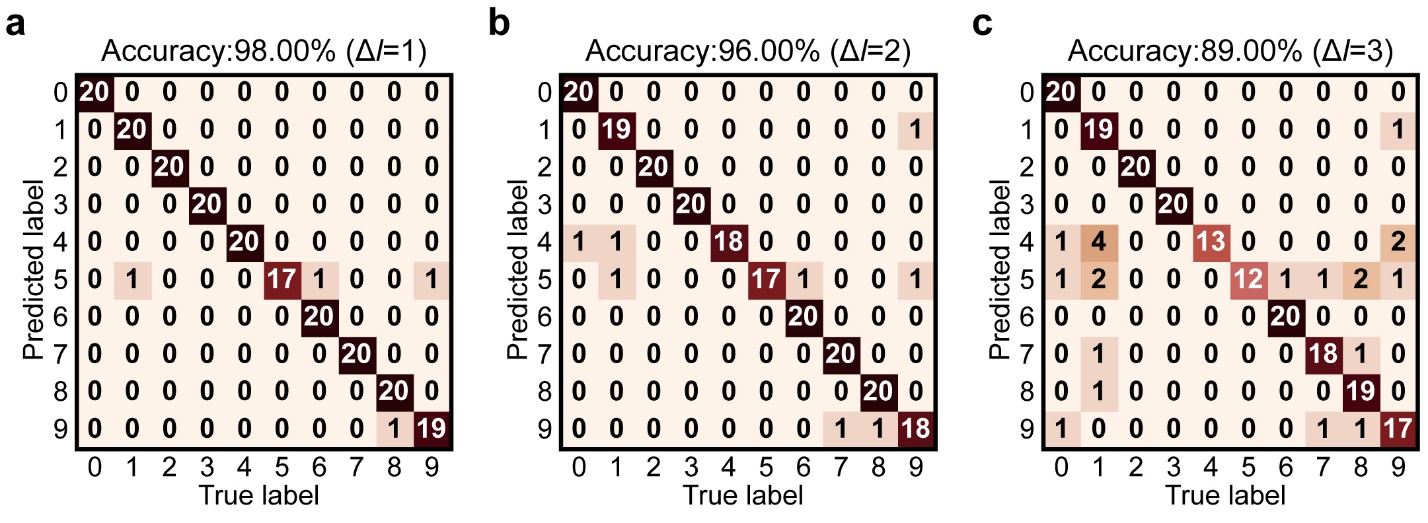


**Fig. S5** **The performances of the CNN with different target encoding OAM states. a-c**. The interval of the encoding OAM orders ranges from 1-3.


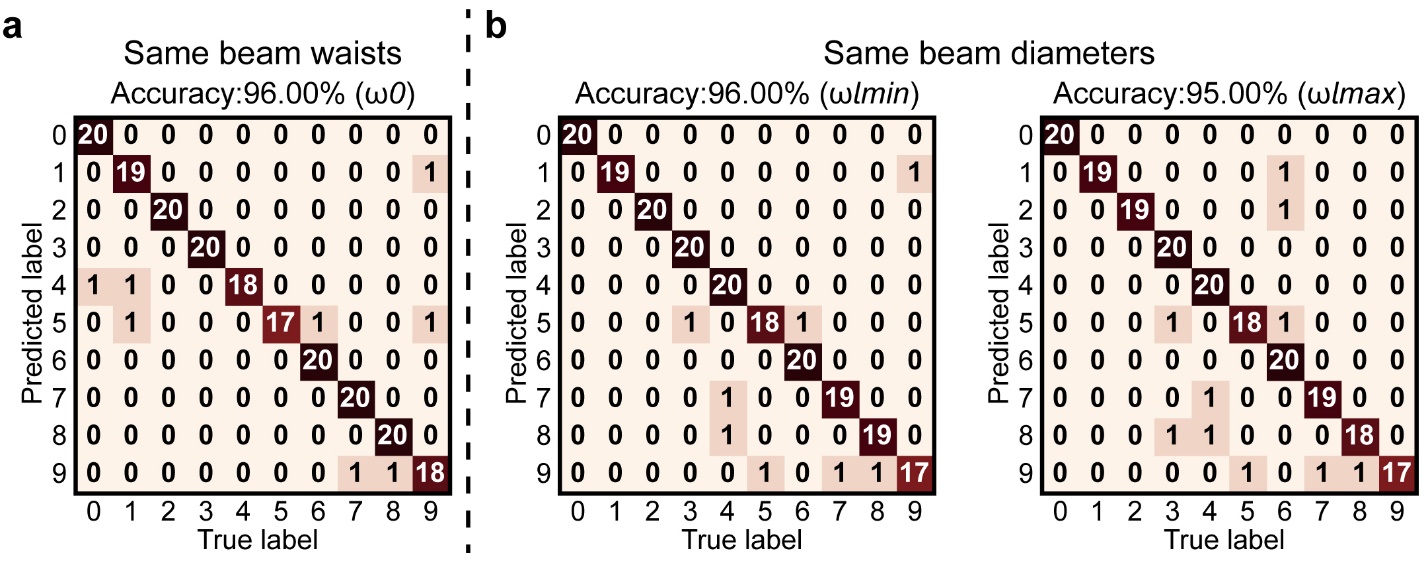


**Fig. S6. The effect of the beam size of the target encoding LG mode basis on the performances of the CNN.** **a**. The encoding LG modes basis with the same beam waists. **b**. The encoding LG modes basis with the same maximum (left panel, determined by *l*=±9) /minimum (right panel, determined by *l*=±1) diameters.


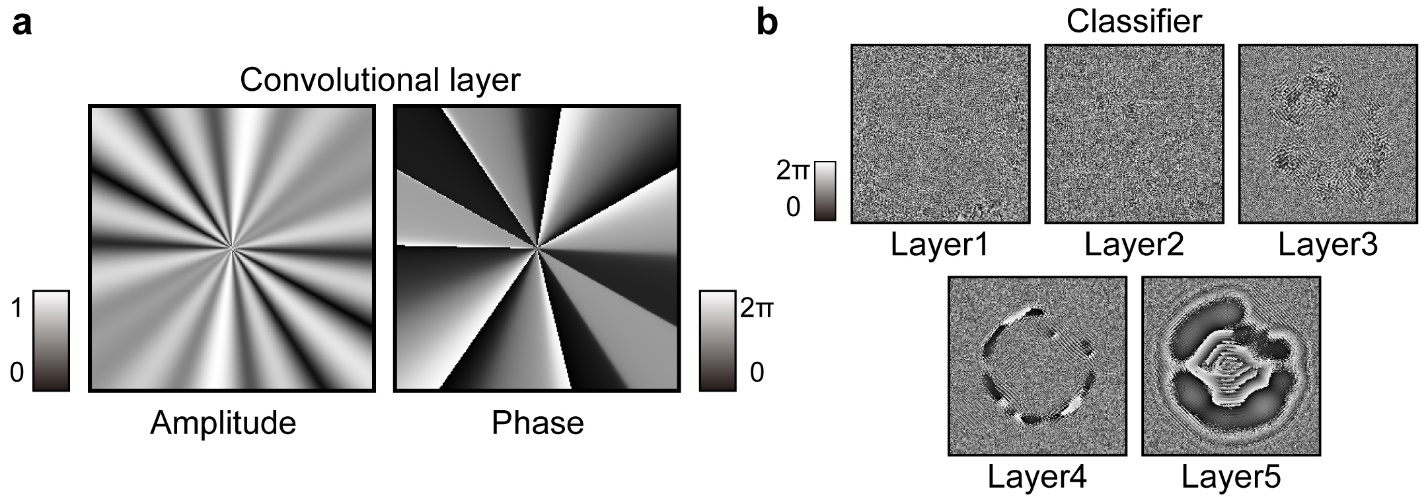


**Fig. S7.** **The amplitude/phase distributions of (a). the convolutional layer and (b). the classifier of the CNN in Fig. 2c.**


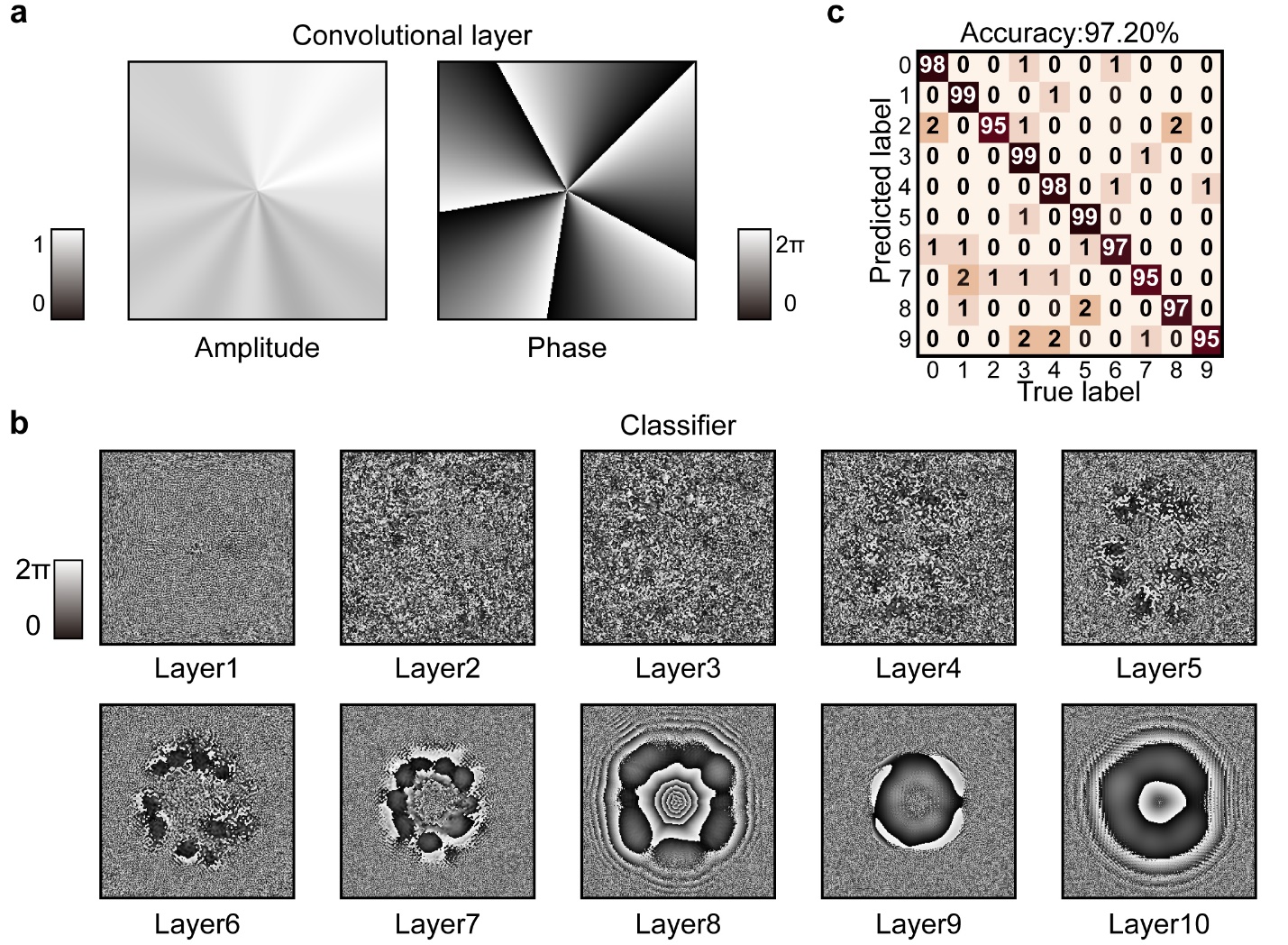


**Fig. S8. The performance of the CNN with a classifier comprising 10 diffractive layers. a,** The amplitude/phase distributions of the convolution layer and classifier. **b,** The phase distributions of the ten-layer classifier. **c,** The confusion matrix with a testing accuracy of 97.2 %.


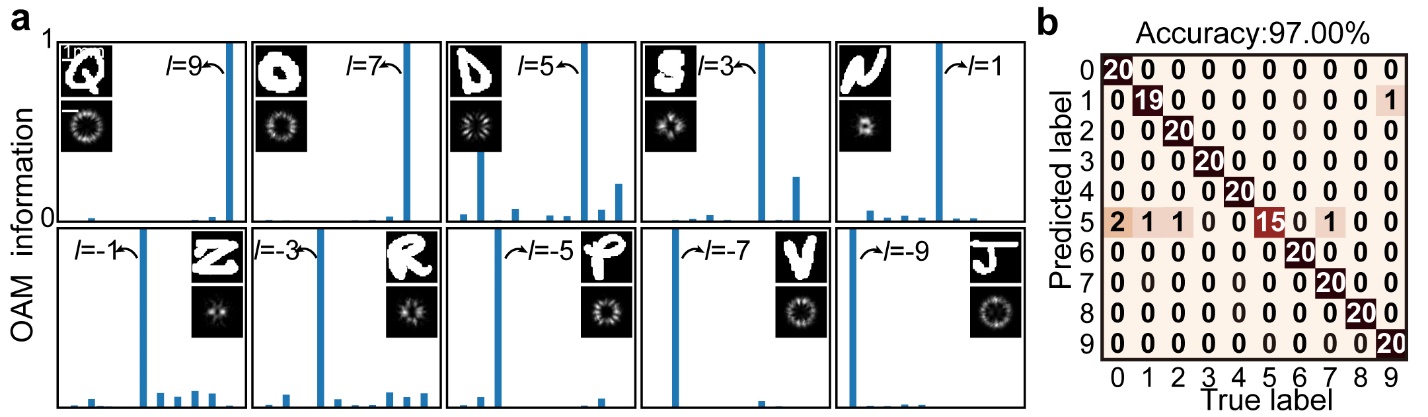


**Fig. S9. Mode-feature encoding with ten classes images within EMINST dataset through CNN. a**. For ten testing images, the intensity distributions and the OAM information are shown, respectively. **b**. The confusion matrix with a testing encoding accuracy of 97.0%.


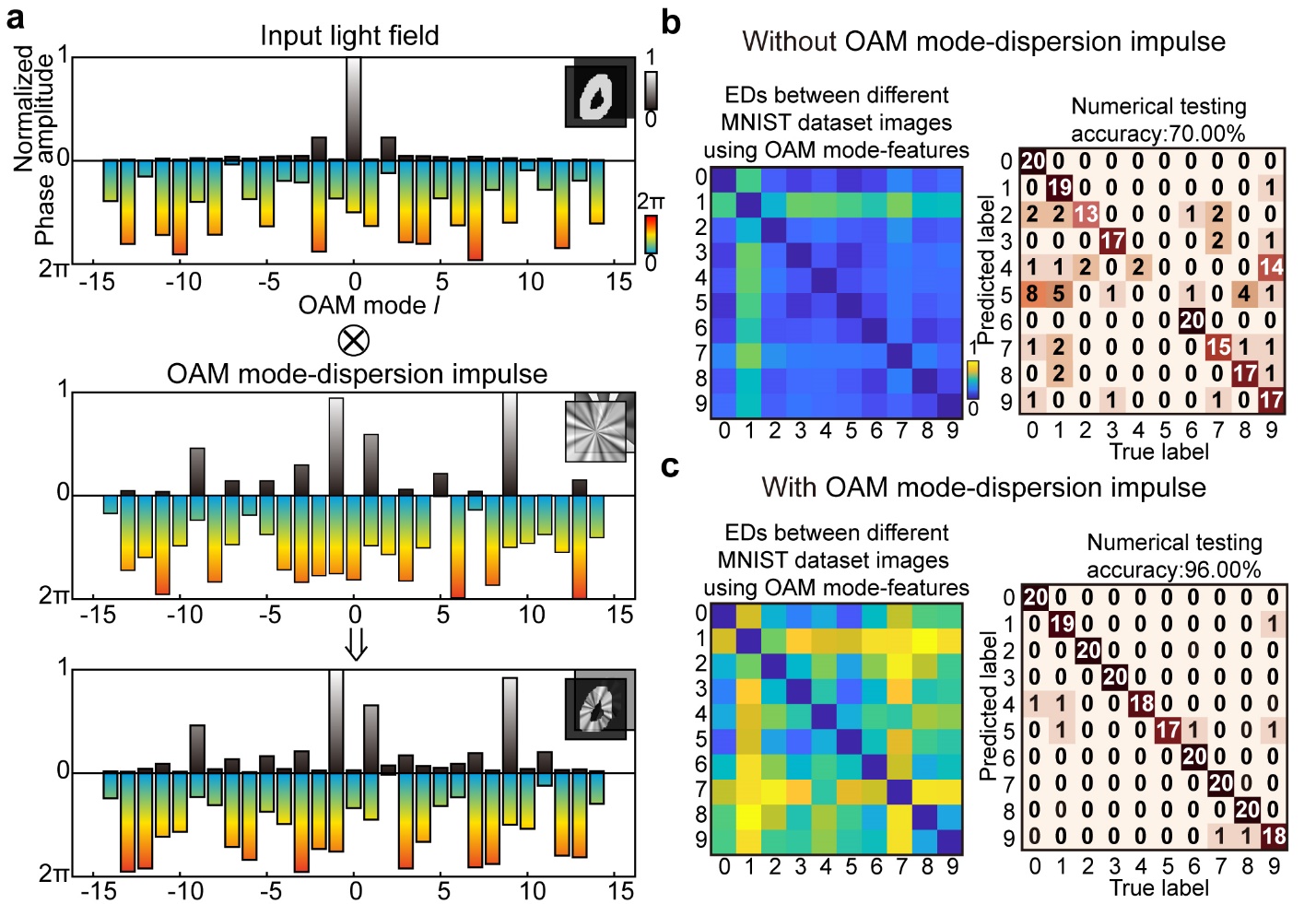


**Fig. S10. Comparison of CNN and DNN in OAM mode-feature encoding. a.** Conversion of OAM mode spectrums (pattern of digital number “0”) after a convolutional operation with an OAM mode-dispersion impulse. **b** and **c.** EDs between ten randomly selected digital images in separate classes with/without convolutional operation, and the confusion matrix of CNN and DNN.

**
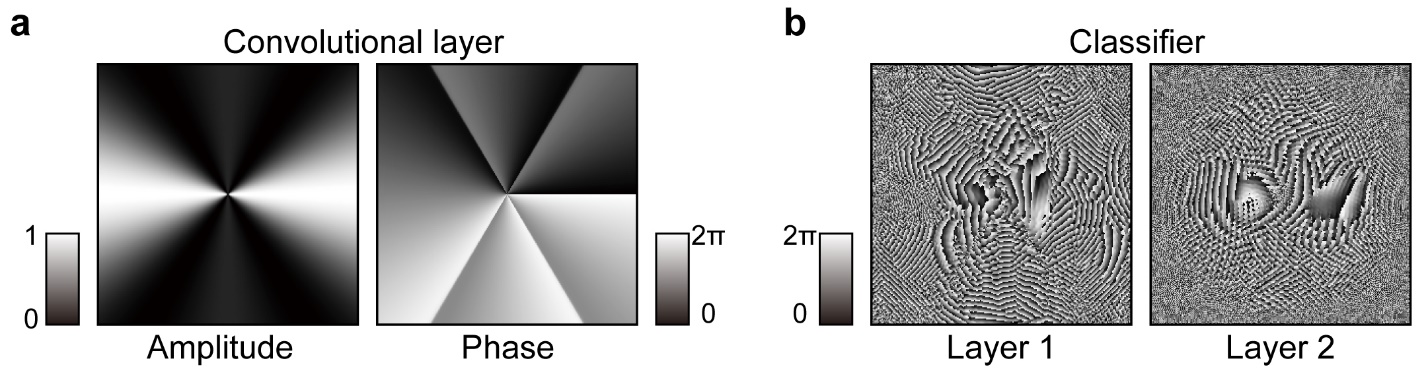
**

**Fig. S11.** **The amplitude/phase distributions of (a) the convolutional layer and (b) the classifier used in Fig. 3.**


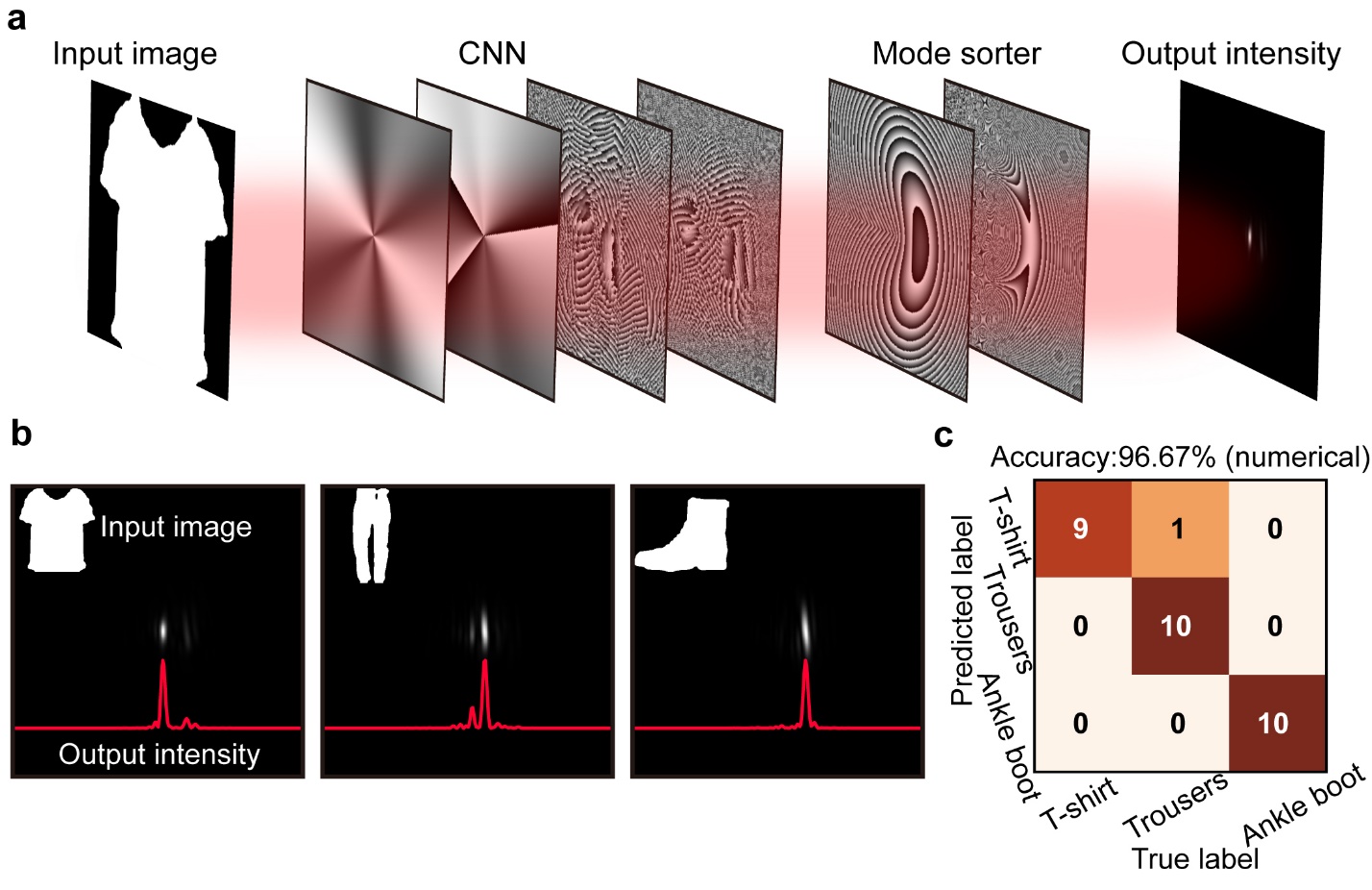


**Fig. S12. The performance of image classification using a mode sorter as the OAM decoder. a**. The phase plates distributions of mode sorter. **b**. The output intensities when different Fashion-MNIST date images pass through the system in a, the images can be identified by the spot locations. **c**. The confusion matrix with a numerical encoding accuracy of 96.67%.

**
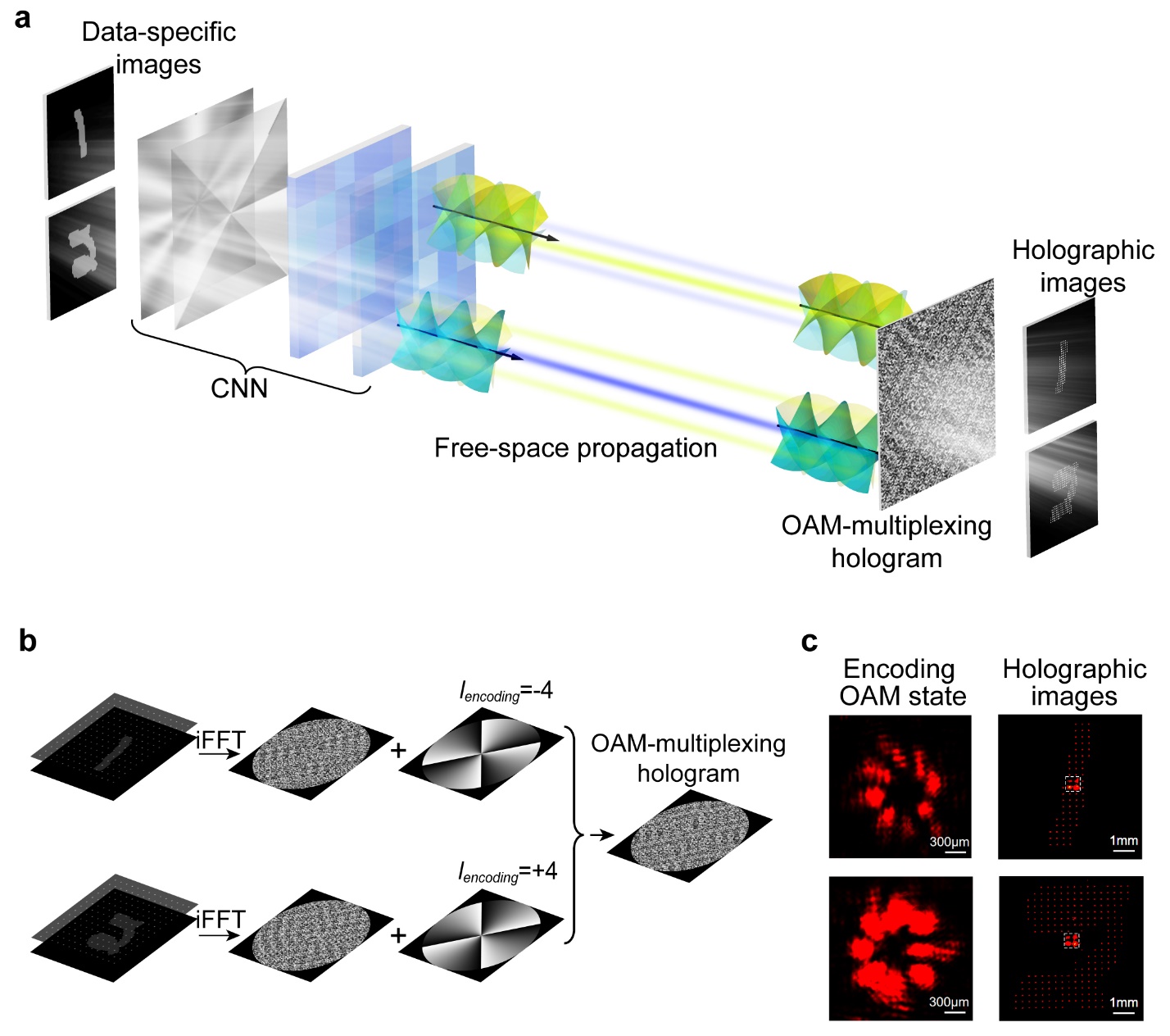
**

**Fig. S13.** **Experimental demonstration of OAM-mediated machine learning for end-to-end switchable image display. a,** Conceptual illustration. **b,** Design of the OAM-multiplexing hologram. **c,** Experimental results of encoding OAM states and holographic images. The spots in the white dashed boxes are generated by the unmodulated light beams of the SLM.


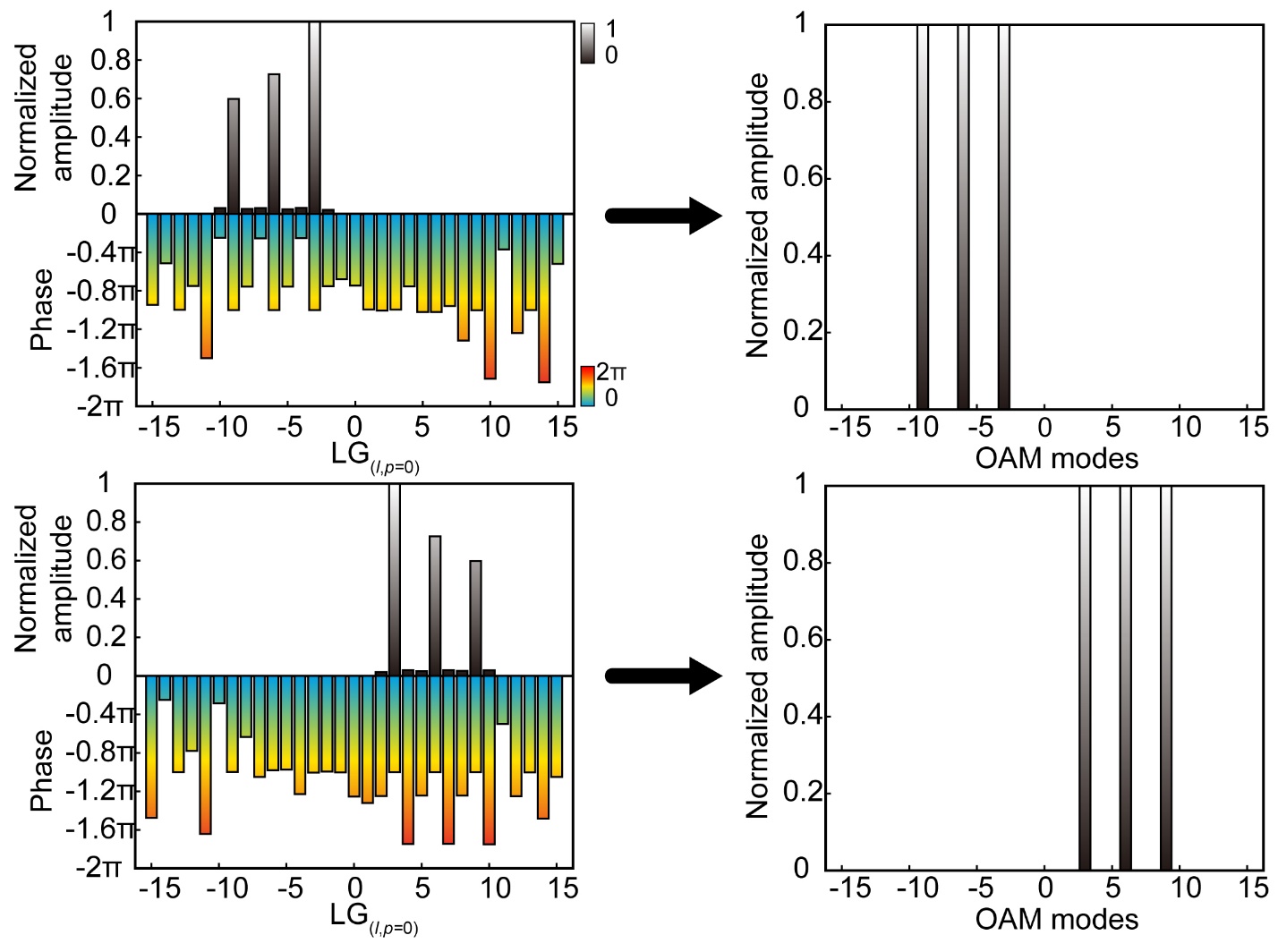


**Fig. S14.** **The complex superposed LG modes with radial index *p*=0 to obtain the target encoded multiplexed OAM mode states (*l*=-9,-6,-3) and *l*=(3,6,9).**


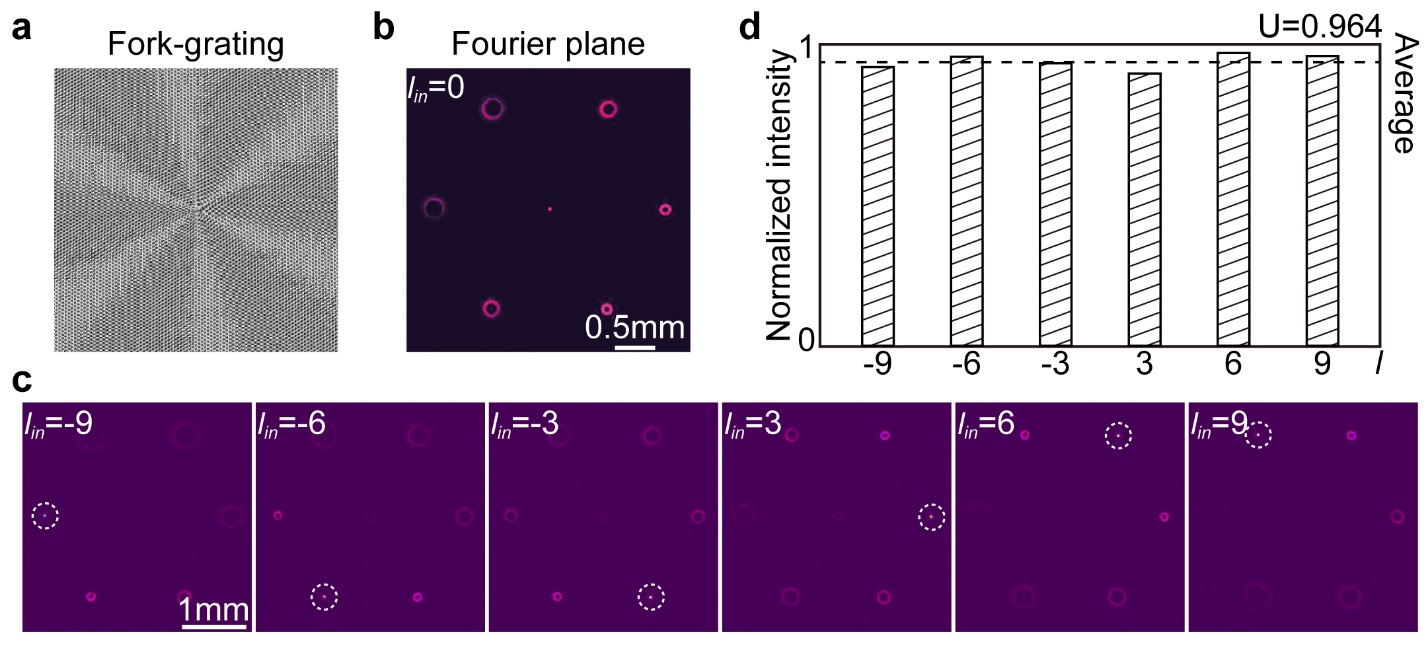


**Fig. S15. Performance of the fork grating in Fig. 4. a,** Phase distribution. Intensity distributions in the Fourier plane when illuminated by (**b)** Gaussian beam and (**c)** the OAM beams with *l* ranging from -9 to 9 with an interval of 3. **d,** Normalized intensity of each diffraction order with different OAM orders *l*.


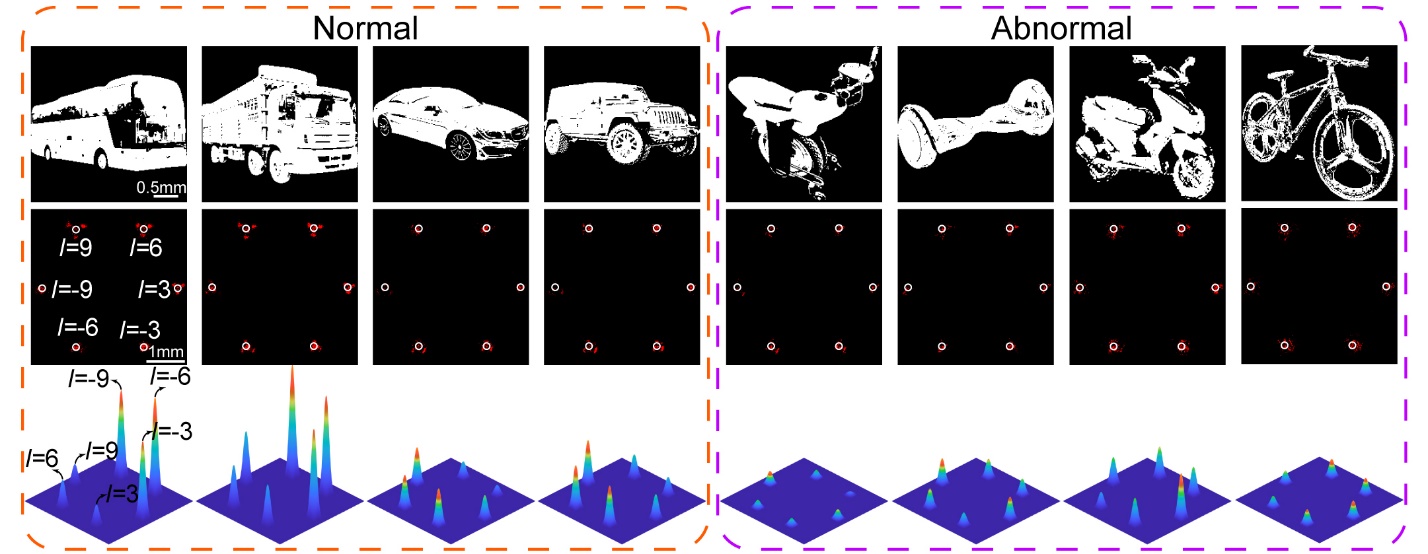


**Fig. S16. The output intensities of the OAM decoder corresponding to the 4 selected normal images and 4 abnormal images in the first low.**
